# Climate change projections of West Nile virus infections in Europe: implications for blood safety practices

**DOI:** 10.1186/s12940-016-0105-4

**Published:** 2016-03-08

**Authors:** Jan C. Semenza, Annelise Tran, Laura Espinosa, Bertrand Sudre, Dragoslav Domanovic, Shlomit Paz

**Affiliations:** European Centre for Disease Prevention and Control, Stockholm,, SE-171 83 Sweden; CIRAD, UPR Animal et Gestion Intégrée des Risques, Montpellier,, F-34093 France; Department of Geography and Environmental Studies, University of Haifa, Mt. Carmel, Haifa,, 31905 Israel

**Keywords:** West Nile fever, West Nile virus, Climate change, Blood safety, Blood supply, Environmental determinants, Epidemiology, Temperature, Surveillance, Arbovirus, Remote sensing, Risk maps

## Abstract

**Background:**

West Nile virus (WNV) is transmitted by mosquitoes in both urban as well as in rural environments and can be pathogenic in birds, horses and humans. Extrinsic factors such as temperature and land use are determinants of WNV outbreaks in Europe, along with intrinsic factors of the vector and virus.

**Methods:**

With a multivariate model for WNV transmission we computed the probability of WNV infection in 2014, with July 2014 temperature anomalies. We applied the July temperature anomalies under the balanced A1B climate change scenario (mix of all energy sources, fossil and non-fossil) for 2025 and 2050 to model and project the risk of WNV infection in the future. Since asymptomatic infections are common in humans (which can result in the contamination of the donated blood) we estimated the predictive prevalence of WNV infections in the blood donor population.

**Results:**

External validation of the probability model with 2014 cases indicated good prediction, based on an Area Under Curve (AUC) of 0.871 (SD = 0.032), on the Receiver Operating Characteristic Curve (ROC). The climate change projections for 2025 reveal a higher probability of WNV infection particularly at the edges of the current transmission areas (for example in Eastern Croatia, Northeastern and Northwestern Turkey) and an even further expansion in 2050. The prevalence of infection in (blood donor) populations in the outbreak-affected districts is expected to expand in the future.

**Conclusions:**

Predictive modelling of environmental and climatic drivers of WNV can be a valuable tool for public health practice. It can help delineate districts at risk for future transmission. These areas can be subjected to integrated disease and vector surveillance, outreach to the public and health care providers, implementation of personal protective measures, screening of blood donors, and vector abatement activities.

**Electronic supplementary material:**

The online version of this article (doi:10.1186/s12940-016-0105-4) contains supplementary material, which is available to authorized users.

## Background

West Nile virus (WNV) is responsible for large outbreaks of fatal neuroinvasive disease worldwide [1]. WNV infections occur predominantly through mosquito bites but also through blood transfusion or organ, tissue and cell-transplantations. Most human infections are asymptomatic and mild cases present only flu-like symptoms; more severe cases present with signs of encephalitis, meningo-encephalitis or meningitis. Globally, WNV is a widespread arthropod-borne virus, an enveloped, single-strand RNA virus of the genus *Flavivirus* in the family of *Flaviviridae* [2–4]. It circulates in Africa, Americas, Asia, Europe, and Australia, where it is thought have been introduced from the Middle East [4]. In Europe, Middle East and Africa, WNV has been responsible for sporadic outbreaks in the 1950s in Israel, in the 1960s in Russia and France, in the 1970s in Belarus, South Africa, and Ukraine. However, since 1996 it has caused more recurrent outbreaks in Europe and northern Africa [5–8]. In 2010, large outbreaks in humans occurred in Southeastern and Eastern Europe. The European outbreaks occurred in Russia, the Czech Republic, Hungary, Romania, Turkey, Greece, Italy, France, Spain and Portugal [9]. Since 2010, there have been annual outbreaks in Southeastern and Eastern Europe, suggesting an endemic transmission cycle and thus a resurgent public health problem [10].

The transmission cycle of WNV is maintained in nature in an enzootic cycle between susceptible birds and competent ornithophilic mosquitos. A number of intrinsic and extrinsic factors are responsible for the complex nature of WNV epidemiology. Intrinsic factors include vector and host competence, genetic traits, mosquito feeding rates and preferences, mosquito longevity and host immunity [11]. WNV has been detected in over 60 species of mosquitoes but in Europe *Culex pipiens* seems to play the most important role [12, 13]. These mosquitoes can act as bridge-vectors that feed both on birds as the principal host as well as on incidental hosts such as humans and horses. Local birds are responsible for WNV amplification while infected migratory birds account for the long-distance dispersion of WNV. The extrinsic factors include abiotic conditions such as the environmental settings that shape the transmission dynamic but also biotic factors such as the density and composition of hosts and vectors [9].

In Europe, there are sylvatic and synanthropic transmission scenarios, in rural and urban areas. They respectively (and independently) contribute to high concentrations of hosts with competent mosquito vectors that support intense local avian transmission. Rural areas with estuaries, wetlands or marshes attract migratory birds for breeding, nesting, and rearing their young. These bird habitats also attract bird-feeding mosquitoes where congregating bird populations can get infected with WNV. In fact, a number of human outbreaks have originated in European estuaries such as in the Danube delta in Romania, in the Volga delta in Russia, and in the Rhone delta in France [14]. Urbanized areas can also attract large bird and mosquito populations where humans can get exposed [15]. Moreover, urban infrastructure can serve as mosquito breeding sites, such as pools in ineffective drainage systems [16]. For example, in 1996, a large outbreak occurred in the city of Bucharest, Romania that affected 4 % of the population [7]. Over 800 patients were hospitalized during another large WNV outbreak that occurred in 1999 in Volgograd City on the west bank of the Great Volga River [17] and in 2010 WNV infections were documented for the first time in humans in the Greek city of Thessaloniki [18, 19]. Certain metropolitan areas can sustain a high breeding density of birds; for example, European Starlings thrive on urban lawns or parks where they can feed, or gulls proliferate near open water [20, 21]. Birds from these types of urban environments harbour viruses with higher genetic diversity than birds from residential areas, indicating that anthropogenic factors associated with urbanization play an important role in arboviral transmission and evolution [22]. In the United States, WNV infection rates in crows and humans are higher in more urbanized environments that are less forested [23, 24], while in Europe, WNV transmission can occur both in rural and urban areas.

Ambient temperature is another important environmental determinant in the transmission of WNV as it has a direct impact on mosquito survival, developmental rates of immature stages, growth rates of vector populations and decrease in the interval between blood meals [25–28]. Moreover, temperature also affects the extrinsic incubation period (the number of days from ingestion to transmission) by influencing the viral replication rates and thus the transmission of WNV [29, 30]. In a modelling study elevated air temperature was the strongest predictor of increased infection in mosquito vectors [28]. As for Europe, it was found that the unprecedented upsurge in the number of human WNF cases in the summer of 2010 was preceded by extremely hot spells in the region [25]. Moreover, recent research analysed the status of infection by WNV in Europe and its neighbouring countries in relation to environmental and climatic risk parameters. The anomalies of temperature in July were identified as one of the main risk factors [10]. During recent decades, parts of Europe have warmed up more than the global average. Additionally, more frequent and more intense hot extremes have occurred. This trend is expected to continue while predictions suggest a further temperature increase (between 1.0 °C and 5.5 °C) by the end of the century [31]. Increases in ambient temperature due to climate change are therefore projected to impact WNV transmission in Europe and its neighbouring countries [32–36].

In order to examine these land-use and climatic variables as predictors of the probability of WNV infection [4] we tested the contribution of remotely sensed temperature, the state of vegetation and water bodies, and bird migratory routes in a statistical model. We also project the WNV risk in Europe into 2025 and 2050, with July temperature projections under a balanced IPCC climate change scenario (A1B) [37]. Among IPCC scenarios that cover a wide range of the main demographic, economic, and technological driving forces of greenhouse gases and sulphur emissions, the A1 scenario groups are distinguished by their technological emphasis. A1B represent a balance across all energy sources (intensive fossil and non-fossil energy). Balanced is defined as not relying too heavily on one particular energy source on the assumption that similar improvement rates apply to all energy supply and end use technologies [37].

Insights from these analyses can also be used to assess the current and future WNV risk to the safety of blood supply in the region [38]. Thus, we provide a quantification of the risk of WNV transmission through blood transfusion by estimating the prevalence of infection in (donor) populations of an area affected by a WNV outbreak, as well as the infection risk of a blood donor that visited an outbreak affected area. In the long-run, the environmental determinants identified in this model lend themselves for an integration of environmental monitoring in public health surveillance systems of human cases, serological surveillance of domestic and wild avifauna, and entomological surveillance [4, 39, 40].

## Methods

### Temperature data

The association between WNV outbreaks in Europe and a number of temperature parameters such as mean, minimum and maximum and their anomalies were examined by Paz et al. (2013) [25]. Positive temperature anomalies were found to be a major risk factor for WNV occurrence. Subsequently, in a study by Tran et al. (2014) remotely sensed mean temperature anomalies of July were identified as a risk factor for WNV transmission in Europe [10]. Based on these observations temperature anomalies were modelled for the present analysis. Monthly anomalies of July 2014 temperatures at the locations of WNF outbreaks reported in humans were computed from NOAA NCEP-NCAR (US National Centre for Atmospheric Research) database. Predicted surface temperatures and anomalies were extracted for 2015–2050 climate change A1B scenario, all for the gridded region 30°N-60°N and 10°W-55°E [41]. The predicted temperatures were based on the Community Climate System Model (CCSM3) of NCAR, generated on a Gaussian grid. Following NCAR methodology, the temperature anomalies data in the current study are relative to 20th Century Experiment 1980–1999 [36]. The A1B Scenario was chosen since it is a balanced scenario; its main characteristics include: low population growth, very high GDP growth, very high energy use, low-medium land use changes, medium resource (mainly oil and gas) availability, rapid pace and direction of technological change favouring balanced development. The chosen model output is the Ensemble Average which is the mean state of the climate among all model runs. Global climate simulations were produced at NCAR by the CCSM3 for the 4th Assessment report of the IPCC [42].

### WNV epidemiology

The methods for the WNV epidemiological model in Europe have been described previously [10]. Briefly, the epidemiologic data of human West Nile cases were obtained from the West Nile fever surveillance conducted at ECDC during the transmission season in Europe [43]. The transmission season in Europe starts in May and extends into November. Population data for the European Union were based on the nomenclature of territorial units for statistics classification (NUTS) at level 3 with population estimate of 2010 [44]. Global Administrative Unit Layers (GAUL) were used for regions outside of the European Union, and project and population estimates for the year 2010 were derived from the Gridded Population of the World (GPW) dataset [45].

### Environmental variables

To derive the Normalized difference vegetation index (NDVI) and Modified normalized difference water index (MNDWI) values, Moderate resolution imaging spectroradiometer (MODIS) data products were acquired from Land Process Distributed Active Archive Center (LP DAAC). MODIS Terra 8-day composite images of surface reflectance estimates at 500 m spatial resolution (product MOD09A1) were acquired for all WNV infected countries for a twelve years period (2002–2013).

Remotely sensed vegetation indices such as the NDVI have been identified as risk factors for WNV outbreaks occurrence in previous North American studies [46, 47]. Indeed, NDVI may serve as an indicator of environmental conditions suitable for vegetation growth and emergence of mosquito populations [46]. The presence of water bodies was identified as another environmental WNV risk factor [47], because large standing water resources may lead to an upsurge of mosquito populations. The Modified Normalized Difference Water Index (MNDWI) is particularly suited to the detection of free water [48]. This index was found to be more appropriate for the analysis than precipitation due to the inconsistent association between precipitation and WNV outbreaks [36]. The presence or absence of wetlands in a district was defined according to Ramsar Sites Information (https://rsis.ramsar.org/) and the Global Lakes and Wetlands Database (GLWD, level data 1 and 2) (http://www.worldwildlife.org/pages/global-lakes-and-wetlands-database). Passerine fly ways were digitized in order to categorize administrative units in two categories of migration flyway (western and eastern) according to the migration flyways of Western Palearctic Passerines South Eastern European bird migration network (http://www.seen-net.eu/).

The long-term average and standard deviation of each of the environmental indices were computed on monthly bases for the temperature data, and on 8-days interval bases for the MODIS NDVI and MNDWI data. The anomaly (*z*) of temperatures, NDVI and MNDWI was calculated for each date *i* (month or 8-days period) as a function of the annual indices *x*_*i*_ and their long-term average and standard deviation values. The mean anomalies of temperatures, NDVI and MNDWI were computed for each district and each month and MODIS 8-days period. The analysis was performed at the district level (*n* = 1113) categorized as ‘infected’ if WNF cases in humans were reported there that year, and as ‘non-infected’ otherwise.

### Multivariate models

We used multivariate logistic regression models to test the status of a district as ‘infected’ or ‘not infected’ as the response variable. Thus, the probability distribution was assumed to be binomial, and the response variable a logit function. As explanatory variables we used the population, the presence of wetlands, the presence of birds’ migratory routes, the anomalies of temperature, NDVI and MNDWI as previously described [10]. We also tested as explanatory variable: the occurrence of a WNV outbreak the previous year (λ: weighted average of the number of infected districts amongst the neighbourhood the previous year), considering that WNV could persist locally through survival in overwintering mosquitoes or infected birds. A bootstrap procedure (1,000 replicates) was applied to estimate the 95 % confidence interval (95 % CI) of the parameter estimates, selecting randomly each time from the original set of 1113 districts 90 % of infected districts between 2002 and 2011 (*n* = 98) and 90 % of non-infected districts (*n* = 903). The final model used in the current analysis is described in Table 1. This model was validated using 2012–2013 epidemiologic data that were not used for model constructions [10].

**Table 1:** Multivariate logistic regression model parameter of the risk of WNV infection at district level, EU and neighbouring countries [10]

	Parameter	95 % CI	*p*-value
Intercept	−5.85	[−6.02;-5.74]	-
TMPJUL	**0.37**	[0.32;0.41]	<10-7
MNDWI21	**1.14**	[1.06;1.22]	<10-15
λ	**5.06**	[4.78;5.31]	<10-15
WETLANDS	**1.38**	[1.16;1.55]	<10-7
Absence			
Presence			
MIGRATION	**1.04**	[0.91;1.24]	<10-7
Western path			
Eastern path			
POPULATION	1.66 10-7	[1.66 10-7;2.21 10-7]	<10-2

Significant variables are highlighted in bold characters

TMPJUL: Monthly anomalies for July temperature from the perennial mean monthly temperature

MNDWI21: 8 days anomalies for June Modified Normalized Difference Water Index

λ: Weighted average of the number of infected districts amongst the neighbourhood the previous year

Migration: Passerine fly ways were dichotomized into western and eastern migration flyways according to the migration flyways of Western Palearctic Passerines South Eastern European bird migration network (http://www.seen-net.eu/)

The average, standard deviation, and anomalies of the 2014 temperature was computed and applied to the model described above [10]. The results were compared with the actual occurrence of WNV in 2014. Validity of the model was assessed based on the ability of the model to distinguish between districts with and without WNV, using sensitivity, specificity, and Area Under Curve (AUC) of the Receiver Operator Characteristic Curve (ROC).

### Climate change projections

The model was used to predict the probability of WNV infection by applying the projected July temperatures for 2025 and 2050. We extracted gridded temperature projections for the A1B Scenario, which is a balanced climate change scenario [42]. July temperature anomalies were entered for each year between 2015 and 2050 in order to compute the probability of WNV infections per district and per year which is one of the parameters in the model (infection the previous year) [10].

### Projections of the prevalence of infection

To assess the safety of the blood supply from a WNV infection we used the European Up-Front Risk Assessment Tool (EUFRAT) developed by ECDC [49]. The risk to transfusion recipients in outbreak affected regions can be computed with this tool, quantifying the risk of transmission of emerging pathogens through blood transfusion. The risk can be calculated for any infectious disease for which a number of characterizing parameters are known or, as in this study, estimated (i.e. population size, prevalence, duration of epidemic, proportion of undetected cases, etc.). We calculated the prevalence of infection in (donor) population in affected areas and the probability of the blood donor to become infected after visiting such an area. For the calculation, the absolute number of donors infected with WNV in the outbreak-affected area was used from the notification/surveillance systems reported to ECDC. This number refers to individuals in the population of the outbreak-affected areas that are reported/ notified as cases when they seek health care during the epidemic. We used 60 % as a conservative estimate of the proportion of undetected cases which includes WNV infected individuals that may not develop symptoms, did not seek for health care, or were misdiagnosed with other diseases and were thus not reported as cases. The population size was entered as the population number in the outbreak-affected areas where the notified cases were recorded. The proportion of chronic infection was set to zero. The duration of the epidemic was the length of period since the first case was reported up until the last day of reported cases and was set to 300 days. The prevalence of infection in the (donor) population was calculated based on these parameters for 2014.

## Results

**Fig. 1:**
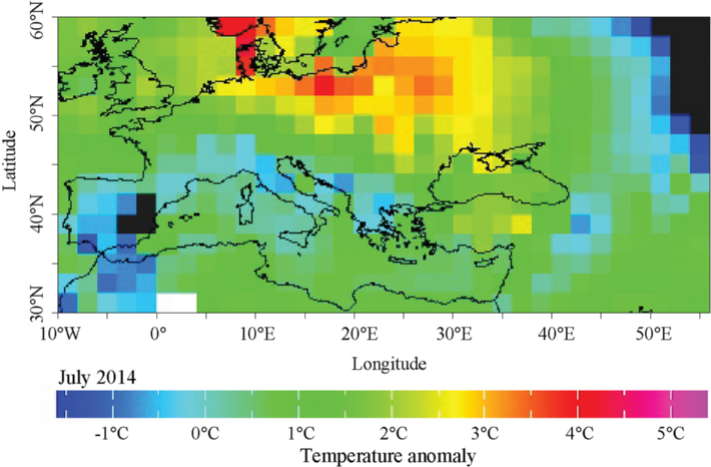
Temperature anomalies for July 2014

**Fig. 2:**
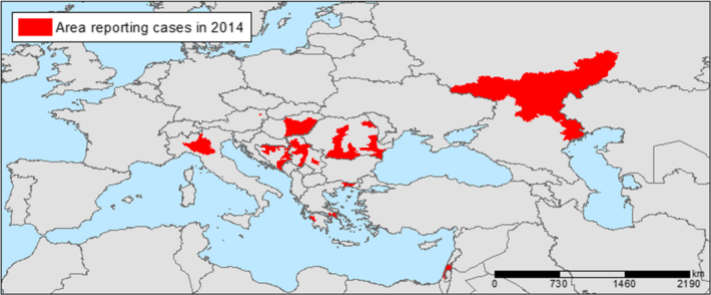
Districts with probable and confirmed cases of West Nile Virus infections, as of 20/11/2014. Note: An affected area is defined as an area with one or more autochthonous human WNV cases (neuro-invasive and non neuro-invasive), meeting laboratory criteria as per EU case definition’ (Directive 2008/426/EC). Probable and confirmed: A probable case is any person meeting the clinical criteria AND with at least one of the following two: − an epidemiological link; − a laboratory test for a probable case. WNV cases by country: Austria (1); Greece (15); Hungary (11); Italy (24); Romania (23); Bosnia and Herzegovina (13); Israel (17); Palestine (1); Russian Federation (29); and Serbia (76)

**Fig. 3:**
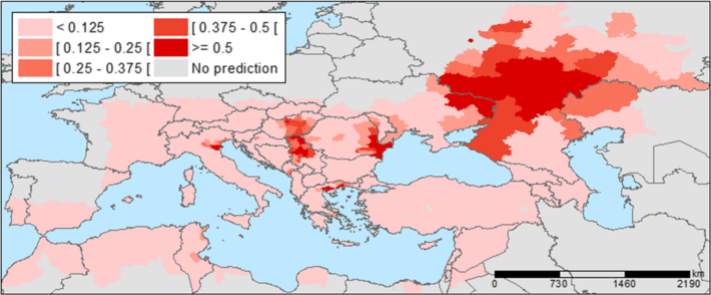
Predicted probability of districts with West Nile Virus infections for 2014

**Fig. 4:**
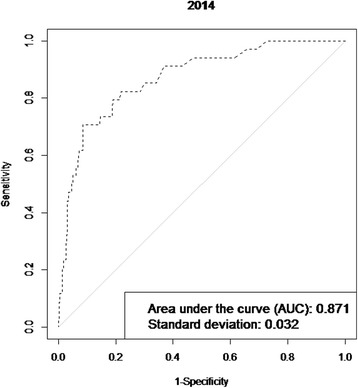
Receiver operating characteristic (ROC) curve of the probability of West Nile Virus infections in 2014

Temperature anomalies in Europe for July 2014 are shown in Fig. 1. The locations of 210 probable and confirmed cases of WNV infections in Europe and neighbouring countries for 2014 are illustrated in Fig. 2. An affected area was defined as an area with one or more autochthonous human WNV cases which were recorded in a number of countries: Austria (1); Greece (15); Hungary (11); Italy (24); Romania (23); Bosnia and Herzegovina (13); Israel (17); Palestine (1); Russian Federation (29); and Serbia (76). We used the multivariate logistic regression model developed by Tran et al. (2014) to compute the probability of WNV occurrence based on July temperatures anomalies, the anomaly of the Modified Normalized Difference Water Index (MNDWI) in early June, an outbreak of the previous year, the size of the human population, wetlands and the type of avian flyways [10]. We applied a standard threshold of 0.5 to classify infected (with probability of infection greater than 0.5) and non-infected (with probability of infection lower than 0.5) districts. The probability map with the July 2014 temperature deviation is presented in Fig. 3. Geographic areas of predicted probability of high WNV infection were North-eastern Greece, central Hungary, North-eastern Italy, Eastern Romania, central Serbia, and large areas of Southern Russia. This probability map was compared with the actual occurrence of WNV cases in 2014 (Fig. 2) and a concordance was observed. Validity of the model was assessed based on the ability of the model to distinguish between districts with and without WNV. External validation of the model with the 2014 data indicated good prediction based on the Receiver Operating Characteristic (ROC) Curve (Fig. 4). The Area Under Curve (AUC) of the model reached 0.871 (SD = 0.032). Sensitivity was calculated as the proportion of WNV positive districts correctly identified by the model and yielded 0.236 (SD = 0.074). Specificity was calculated as the proportion of WNV negative districts correctly identified by the model and yielded 0.982 (SD = 0.006).

**Fig. 5:**
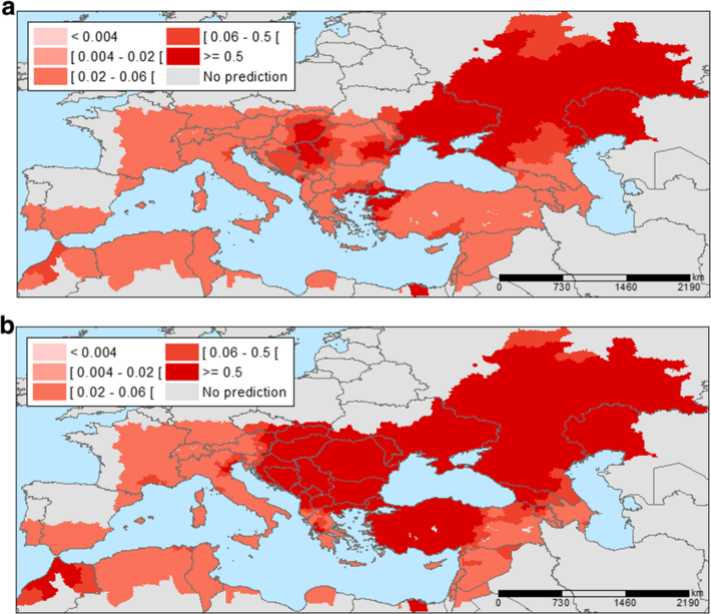
Predicted probability of districts with West Nile Virus infections based on July temperatures for A1B scenario projections for 2025 (**a**) and 2050 (**b**). Note: Among IPCC scenarios, the A1 scenario groups are distinguished by their technological emphasis. A1B represent a balance across all energy sources (intensive fossil and non-fossil energy)

**Fig. 6:**
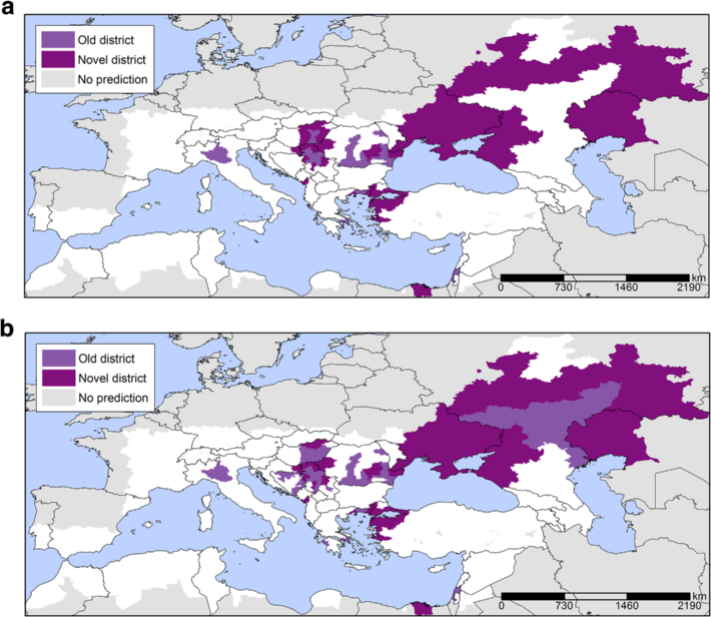
New districts affected by West Nile Virus infections in 2025 compared to 2014. Note: Panel **a**: Confirmed: A confirmed case is any person meeting laboratory criteria for case confirmation. Panel **b**: Total (confirmed and probable): A probable case is any person meeting the clinical criteria AND with at least one of the following two: − an epidemiological link; − a laboratory test for a probable case

**Fig. 7:**
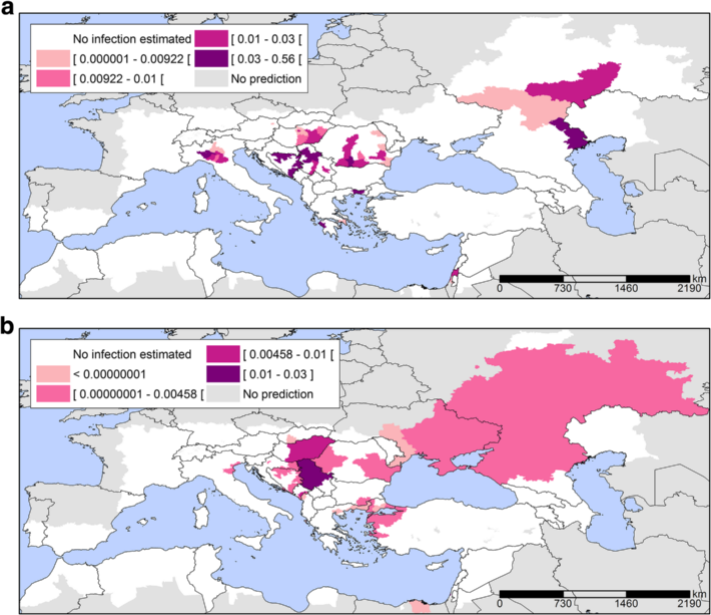
Estimated prevalence of West Nile Virus infections in the blood donor population (per 100,000) by districts for 2014 (**a**) and for 2025 (**b**). Note: The prevalence of infection in the (donor) population was calculated based on the European Up-Front Risk Assessment Tool (EUFRAT) developed by ECDC [49]

Probability maps were generated with the A1B temperature projections (Fig. 5). The results reveal a progressive expansion of areas with an elevated probability for WNV infections, particularly at the edges of the transmission areas. For example, the 2025 map reveals a higher probability of WNV infection in Northern Serbia, Central Hungary, North-Eastern Greece, Eastern and Western parts of Romania and Northwestern Turkey. These are also the areas that recorded new districts affected by WNV infections in 2025 compared to 2014 (Fig. 6), which indicates the expansion of the epidemic. According to this analysis, total of 81 and 268 new districts recorded WNV infections for the first time in 2025 and 2050, respectively compared to 2014 that were situated on the perimeter of the transmission areas. In 2050, the area with a higher probability will have expanded even more with a total of 147 and 405 districts being affected in 2025 and 2050, respectively. WNV infections are a significant concern to the safety of the blood supply, because the blood donated by asymptomatic carriers might inadvertently contaminate the blood supply [38]. Therefore, we calculated the prevalence of infection in (donor) populations in the outbreak-affected areas. We mapped the donor population infectivity (Fig. 7) and found an extended area of elevated WNV infection hazard for the safety of the blood supply in 2025 compared to 2014.

## Discussion

### Projections of WNV risk

The multivariate model for WNV outbreaks developed for the period 2002 to 2011 and validated with 2012 and 2013 data [10] was applied here to July 2014 temperature anomalies. The probability map revealed a good agreement with the actual WNV outbreaks in 2014, based on the ROC curve. Under the A1B climate change scenario for 2025, this model projects a higher probability of WNV infection in Northern Serbia, Central Hungary, North-Eastern Greece, Eastern and Western parts of Romania, and Northwestern Turkey. These are also the areas where new districts will be affected by WNV infections.

The ability of the model to detect a district with WNV infections was moderate (sensitivity = 0.236) while the ability of the model to correctly detect a negative district was very good (specificity = 0.982). The probability cut off was chose based on public health grounds. For example, the threshold value obtained using the observed prevalence method (*p* = 0.07) would generate a sensitivity of 0.706 and a specificity of 0.915. Instead we opted for a higher specificity, which implies (given the compromise Se/Sp) a low sensitivity. In that sense, our predictions are rather optimistic. By increasing the sensitivity the number of WNV positive districts would have increased, potentially “overestimating” the number of WNV positive districts. The distribution of WNV positive districts does not follow a Gaussian distribution, but is skewed to the right. Consequently, a small change in the threshold would translate into a significant increase in the number of WNV positive districts even with potentially few WNV cases per district (It is important to note that the model predicts the probability for a district to be positive for WNV, but not the number of cases). In light of limited resources in public health we chose a more conservative approach trying to identify those districts that are predicted to be truly negative for WNV (high specificity).

These findings indicate that July temperature anomalies can be used in our prediction model as an early warning of the coming WNV season and help delineate areas of imminent transmission. Positive temperature anomalies may lead to an upsurge in the growth rates of vector populations, a decrease in the interval between blood meals, a shortening of the incubation time in mosquitoes, virus evolution rate accelerating and an increase of the viral transmission efficiency to birds [9, 29, 36]. These areas can then be targeted for enhanced epidemiologic surveillance of neuro-invasive illness (which may be suggestive of WNV infection), awareness rising among healthcare workers for the clinical presentation of WNV infection, and reinforced laboratory diagnostic capacity. Moreover, these areas can be subjected to entomological surveillance to characterize mosquito breeding sites, and vector abatement measures to diminish mosquito densities. Passive surveillance in domestic birds and equine populations to monitor the dispersion of WNV might also be considered.

This model was also applied to July temperature projections for 2025 and 2050 in order to quantify the future burden of WNV. These results indicate that 81 and 268 districts will be impacted by WNV outbreaks by 2025 and 2050, respectively. Districts adjacent to districts with current transmission are at elevated risk and should therefore also be considered for the public health interventions listed above.

### Blood supply safety

The arrival and dispersal of tropical pathogens to Europe and its neighbouring countries commonly associated with warmer temperatures pose a threat to the supply of safe blood products, particularly if they are unknown or without diagnostic tests [38, 50]. Thus, emerging infectious diseases will continue to pose a threat to transfusion safety on European and international levels [51–53]. Specifically, a progressive expansion of areas with an elevated probability for WNV infections will increase the threat to the safety of blood transfusion. In geographically larger areas affected by WNV, a higher number of blood donors will be exposed to infection for a longer time period (if the duration of the annual mosquito activity season will be prolonged). Our climate change projections of the predicted probability of WNV infection in Europe have far reaching implications for public health in the future, because the findings in this paper can contribute to WNV preparedness activities. Besides the cases of primary WNV infections, secondary infections through contaminated blood products are of increasing concern to threaten the safety of the blood supply [38]. The asymptomatic blood-borne phase of a WNV infection increases the potential for transmission by transfusion, even if it is relatively short, compared to Hepatitis B virus or HIV [54]. Moreover, WNV has the ability to survive and persist in collected blood and stored blood components and subsequently cause an infection through the intravenous application. Thus, we calculated the prevalence of infection in the donor populations in the outbreak-affected areas (Fig. 7). However, this instantaneous estimate may underestimate the true prevalence of infection if the timing of the WNV epidemic is at the peak of the epidemic curve. Nevertheless, the map reveals considerable vulnerabilities in Southeastern Europe when it comes to the safety of the blood supply. These insights can help transfusion service and clinical staff identify, manage and plan for transfusion-transmitted infections. The overall management of blood safety should be addressed at the institutional level, specifically at regulatory agencies or professional organizations [54]. To preserve the number of eligible donors and an adequate blood supply, authorities will inevitably reassess risk reduction interventions [38]. These include deferral strategies [55, 56], screening strategies and triggers [57–59], and also pathogen reduction technologies [38, 60–62]. Moreover, it might be necessary to distribute blood components to outbreak areas from unaffected areas in order to prevent intermittent shortfalls in the blood supply [63]. Such coordination requires supra-national inventories of blood products that can be dispatched upon demand. Therefore, the projected temperature change with elevated probability for WNV infections and possible increased prevalence of WNV infected blood donors should be taken into account in developing preparedness plans for the WNV safety of the blood supply. This could substantially increase the costs of blood transfusion therapy. The projections presented here may therefore offer an insight into future developments in the risk of transfusion from WNV infections; it also provides an opportunity to timely define the optimal strategy of blood safety in the face of limited available resources.

### Limitations

The projections presented in this paper are computed on the assumption that the other variables remained stable. For example, the population size was maintained constant in these projections; however the contribution of the population size to the model output is very minimal, based on a low parameter value (1.66 10^−7^). Moreover, the Eastern bird migration path was retained in the model, as well as the presence of wetlands, and the MNDWI21 under the assumption that they will not change significantly. Yet, other WNV determinants that were not part of our model could change over the given time period. For example, mosquito control measures could differ at the area and country level and change over time, with implications for WNV incidence. Similarly, the quality of surveillance clinical case detection might differ too and change over time. WNV might spread at different rates in local, amplifying bird populations that might eventually develop herd immunity. Moreover, other environmental determinates might vary over time as well. We used a statistical approach which involved modelling the 2002–2013 WNV distribution to assess the eco-climatic constraints influencing WNV transmission. The advantage of this statistical approach is that the most significant temperature and environmental conditions for determining WNV presence and absence can be identified. In contrast, biological, mechanistic, or deterministic models compute the basic reproduction rate (R_0_) with biological thresholds relevant to WNV transmission. However, parameterization of these biological processes has proven to be difficult; moreover, biological models do not explicitly model the socioeconomic and public health contexts in which WNV transmission occurs. Although there are a number of limitations to our model, its validation performed rather well based on the sensitivity and specificity calculations for WNV infections in 2012, 2013 [10] and 2014.

## Conclusion

In several countries of Southeastern Europe, WNV transmission is now established. Our predictive model suggests further WNV dispersal in the coming years to adjacent districts. Monitoring and modelling climatic and environmental conditions permissive for the interaction of migratory birds, resident birds, competent mosquito vectors and humans can help delineate districts at risk of transmission. In light of the rapid pace of urbanization internationally [64], areas at risk of current and future WNV transmission can be targeted for integrated surveillance, vector control measures, outreach to the public and health care sector, strengthened laboratory capacity for reliable WNV diagnosis, and systematic screening of blood donors [40]. These activities call for inter-sectorial collaboration to tackle the challenges of WNV transmission.

## Additional file

Additional file 1:
**Peer review reports.** (PDF 743 kb)

## References

[1] Petersen LR, Brault AC, Nasci RS (2013). West Nile virus: review of the literature. JAMA.

[2] Ciota AT, Kramer LD (2013). Vector-virus interactions and transmission dynamics of West Nile virus. Viruses.

[3] May FJ, Davis CT, Tesh RB, Barrett AD (2011). Phylogeography of West Nile virus: from the cradle of evolution in Africa to Eurasia, Australia, and the Americas. J Virol.

[4] Ozdenerol E, Taff GN, Akkus C (2013). Exploring the spatio-temporal dynamics of reservoir hosts, vectors, and human hosts of West Nile virus: a review of the recent literature. Int J Environ Res Public Health.

[5] Hayes C, Monath TP (1989). West Nile Fever. The Arboviruses: Epidemiology and Ecology.

[6] Karabatsos N (1985). International Catalogue of Arbovirus Including Certain Other Viruses of Vertebrate.

[7] Tsai TF, Popovici F, Cernescu C, Campbell GL, Nedelcu NI (1998). West Nile encephalitis epidemic in southeastern Romania. Lancet.

[8] Lvov DK, Butenko AM, Gromashevsky VL, Larichev VP, Gaidamovich SY, Vyshemirsky OI (2000). Isolation of two strains of West Nile virus during an outbreak in southern Russia, 1999. Emerg Infect Dis.

[9] Paz S, Semenza JC (2013). Environmental drivers of West Nile fever epidemiology in Europe and Western Asia--a review. Int J Environ Res Public Health.

[10] Tran A, Sudre B, Paz S, Rossi M, Desbrosse A, Chevalier V (2014). Environmental predictors of West Nile fever risk in Europe. Int J Health Geogr.

[11] Hardy JL, Houk EJ, Kramer LD, Reeves WC (1983). Intrinsic factors affecting vector competence of mosquitoes for arboviruses. Annu Rev Entomol.

[12] Gomes B, Sousa CA, Vicente JL, Pinho L, Calderon I, Arez E (2013). Feeding patterns of molestus and pipiens forms of Culex pipiens (Diptera: Culicidae) in a region of high hybridization. Parasit Vectors.

[13] Molaei G, Andreadis TG, Armstrong PM, Anderson JF, Vossbrinck CR (2006). Host feeding patterns of Culex mosquitoes and West Nile virus transmission, northeastern United States. Emerg Infect Dis.

[14] Hubalek Z, Halouzka J (1999). West Nile fever--a reemerging mosquito-borne viral disease in Europe. Emerg Infect Dis.

[15] Deichmeister JM, Telang A (2011). Abundance of West Nile virus mosquito vectors in relation to climate and landscape variables. J Vector Ecol.

[16] Epstein PR (2001). West Nile virus and the climate. J Urban Health.

[17] Platonov AE, Shipulin GA, Shipulina OY, Tyutyunnik EN, Frolochkina TI, Lanciotti RS (2001). Outbreak of West Nile virus infection, Volgograd Region, Russia, 1999. Emerg Infect Dis.

[18] Danis K, Papa A, Papanikolaou E, Dougas G, Terzaki I, Baka A, et al. Ongoing outbreak of West Nile virus infection in humans, Greece, July to August 2011. Euro Surveill. 2011;16(34). 21903037

[19] Danis K, Papa A, Theocharopoulos G, Dougas G, Athanasiou M, Detsis M (2011). Outbreak of West Nile virus infection in Greece, 2010. Emerg Infect Dis.

[20] Savard JL, Clergeau P, Mennechez G (2000). Biodiversity concepts and urban ecosystems. Landsc Urban Plan.

[21] Hodgson JC, Spielman A, Komar N, Krahforst CF, Wallace GT, Pollack RJ (2001). Interrupted blood-feeding by Culiseta melanura (Diptera: Culicidae) on European starlings. J Med Entomol.

[22] Bertolotti L, Kitron UD, Walker ED, Ruiz MO, Brawn JD, Loss SR (2008). Fine-scale genetic variation and evolution of West Nile Virus in a transmission “hot spot” in suburban Chicago, USA. Virology.

[23] LaDeau SL, Calder CA, Doran PJ, Marra PP (2011). West Nile Virus impacts in american crow populations are associated with human land use and climate. Ecol Res.

[24] Brown H, Duik-Wasser M, Andreadis T, Fish D (2008). Remotely-sensed vegetation indices identify mosquito clusters of West Nile virus vectors in an urban landscape in the northeastern United States. Vector Borne Zoonotic Dis.

[25] Paz S, Malkinson D, Green MS, Tsioni G, Papa A, Danis K (2013). Permissive summer temperatures of the 2010 European West Nile fever upsurge. PLoS One.

[26] Paz S, Albersheim I (2008). Influence of warming tendency on Culex pipiens population abundance and on the probability of West Nile fever outbreaks (Israeli Case Study: 2001–2005). Ecohealth.

[27] Meyer RP, Hardy JL, Reisen WK (1990). Diel changes in adult mosquito microhabitat temperatures and their relationship to the extrinsic incubation of arboviruses in mosquitoes in Kern County, California. J Med Entomol.

[28] Ruiz MO, Chaves LF, Hamer GL, Sun T, Brown WM, Walker ED (2010). Local impact of temperature and precipitation on West Nile virus infection in Culex species mosquitoes in northeast Illinois, USA. Parasit Vectors.

[29] Reisen WK, Fang Y, Martinez VM (2006). Effects of temperature on the transmission of west nile virus by Culex tarsalis (Diptera: Culicidae). J Med Entomol.

[30] Andrade CC, Maharaj PD, Reisen WK, Brault AC (2011). North American West Nile virus genotype isolates demonstrate differential replicative capacities in response to temperature. J Gen Virol.

[31] EEA. Climate change, impacts and vulnerability in Europe 2012. An indicator-based report. No 12/2012. 2012. http://www.ecdc.europa.eu/en/healthtopics/west_nile_fever/west-nile-fever-maps/pages/index.aspx.

[32] Greer A, Ng V, Fisman D (2008). Climate change and infectious diseases in North America: the road ahead. CMAJ.

[33] Platonov AE, Fedorova MV, Karan LS, Shopenskaya TA, Platonova OV, Zhuravlev VI (2008). Epidemiology of West Nile infection in Volgograd, Russia, in relation to climate change and mosquito (Diptera: Culicidae) bionomics. Parasitol Res.

[34] Morin CW, Comrie AC (2010). Modeled response of the West Nile virus vector Culex quinquefasciatus to changing climate using the dynamic mosquito simulation model. Int J Biometeorol.

[35] Morin CW, Comrie AC (2013). Regional and seasonal response of a West Nile virus vector to climate change. Proc Natl Acad Sci U S A.

[36] Paz S. Climate change impacts on West Nile virus transmission in a global context. Philos Trans R Soc Lond B Biol Sci. 2015;370(1665). doi:10.1098/rstb.2013.0561. 10.1098/rstb.2013.0561 PMC4342965 25688020

[37] IPCC. Special Report – Emission Scenarios. 2000. Summary for policymakers: https://www.ipcc.ch/pdf/special-reports/spm/sres-en.pdf.

[38] Semenza JC, Domanović D (2013). Blood supply under threat. Nature Climate Change.

[39] Kwan JL, Park BK, Carpenter TE, Ngo V, Civen R, Reisen WK (2012). Comparison of enzootic risk measures for predicting West Nile disease, Los Angeles, California, USA, 2004–2010. Emerg Infect Dis.

[40] Semenza JC, Zeller H. Integrated surveillance for prevention and control of emerging vector-borne diseases in Europe. Euro Surveill. 2014;19(13). 10.2807/1560-7917.es2014.19.13.20757 24721535

[41] NCAR Climate change scenarios: https://gisclimatechange.ucar.edu/.

[42] Pachauri RKaR, A. (Eds.). Contribution of Working Groups I, II and III to the Fourth Assessment Report of the Intergovernmental Panel on Climate Change. 2007. http://www.ipcc.ch/publications_and_data/publications_ipcc_fourth_assessment_report_synthesis_report.htm.

[43] European Center for Disese Prevention and Control (ECDC). West Nile fever maps. 2014. http://www.ecdc.europa.eu/en/healthtopics/west_nile_fever/west-nile-fever-maps/pages/index.aspx.

[44] EUROSTAT. Nomenclature of territorial units for statistics. 2014. http://www.ecdc.europa.eu/en/healthtopics/west_nile_fever/west-nile-fever-maps/pages/index.aspx.

[45] NASA. Socioeconomic Data and Application Centre (SEDAC): Gridded population of the world (gpw), v3. 2014. http://www.ecdc.europa.eu/en/healthtopics/west_nile_fever/west-nile-fever-maps/pages/index.aspx

[46] Chuang TW, Wimberly MC (2012). Remote sensing of climatic anomalies and West Nile virus incidence in the northern Great Plains of the United States. PLoS One.

[47] Leblond A, Sandoz A, Lefebvre G, Zeller H, Bicout DJ (2007). Remote sensing based identification of environmental risk factors associated with West Nile disease in horses in Camargue, France. Prev Vet Med.

[48] Ward MP (2009). Equine West Nile virus disease occurrence and the Normalized Difference Vegetation Index. Prev Vet Med.

[49] ECDC European Up-Front Risk Assessment Tool (EUFRAT) http://eufrattool.ecdc.europa.eu/.

[50] Semenza JC, Sudre B, Miniota J, Rossi M, Hu W, Kossowsky D (2014). International dispersal of dengue through air travel: importation risk for Europe. PLoS Negl Trop Dis.

[51] Dodd RY, Leiby DA (2004). Emerging infectious threats to the blood supply. Annu Rev Med.

[52] Alter HJ, Stramer SL, Dodd RY (2007). Emerging infectious diseases that threaten the blood supply. Semin Hematol.

[53] Stramer SL (2014). Current perspectives in transfusion-transmitted infectious diseases: emerging and re-emerging infections. ISBT Sci Ser.

[54] Stramer SL, Hollinger FB, Katz LM, Kleinman S, Metzel PS, Gregory KR (2009). Emerging infectious disease agents and their potential threat to transfusion safety. Transfusion.

[55] Orton SL, Stramer SL, Dodd RY (2006). Self-reported symptoms associated with West Nile virus infection in RNA-positive blood donors. Transfusion.

[56] Lieshout-Krikke RW, Zaaijer HL, Prinsze FJ (2013). The yield of temporary exclusion of blood donors, exposed to emerging infections abroad. Vox Sang.

[57] Busch MP, Caglioti S, Robertson EF, McAuley JD, Tobler LH, Kamel H (2005). Screening the blood supply for West Nile virus RNA by nucleic acid amplification testing. N Engl J Med.

[58] Kleinman SH, Williams JD, Robertson G, Caglioti S, Williams RC, Spizman R (2009). West Nile virus testing experience in 2007: evaluation of different criteria for triggering individual-donation nucleic acid testing. Transfusion.

[59] Custer B, Tomasulo PA, Murphy EL, Caglioti S, Harpool D, McEvoy P (2004). Triggers for switching from minipool testing by nucleic acid technology to individual-donation nucleic acid testing for West Nile virus: analysis of 2003 data to inform 2004 decision making. Transfusion.

[60] Gallian P, Vignoli C, Dombey AM, Mayaudon V, Lin L, Galichet V (2006). Inactivation of a European strain of West Nile virus in single- donor platelet concentrate using the INTERCEPT blood system. Vox Sang.

[61] Vanlandingham DL, Keil SD, Horne KM, Pyles R, Goodrich RP, Higgs S (2013). Photochemical inactivation of chikungunya virus in plasma and platelets using the Mirasol pathogen reduction technology system. Transfusion.

[62] Burnouf T, Chou ML, Cheng LH, Li ZR, Wu YW, El-Ekiaby M (2013). Dengue virus inactivation by minipool TnBP/Triton X-45 treatment of plasma and cryoprecipitate. Vox Sang.

[63] Bambrick HJ, Woodruff RE, Hanigan IC. Climate change could threaten blood supply by altering the distribution of vector-borne disease: an Australian case-study. Glob Health Action. 2009;2. doi:10.3402/gha.v2i0.2059. 10.3402/gha.v2i0.2059 PMC2802100 20052315

[64] Xinhu L, Jinchao S, Tao L, Dixon J, Guoqin Z, Hong Y. Urbanization and health in China, thinking at the national, local and individual levels. Environ Health. 2016;15(Suppl 1):xx. 10.1186/s12940-016-0104-5 PMC4895783 26961780

