# Supplementary material for: Climate change projections of West Nile virus infections in Europe: implications for blood safety practices
Source: Environ Health. 2016 Mar 8;15(Suppl 1):28. doi: 10.1186/s12940-016-0105-4 (PMC4895699; doi:10.1186/s12940-016-0105-4)

Reviewer 1

Climate Change Projections of West Nile Virus Infections in Europe: Implications for Blood Safety Practices

Authors: Jan C. Semenza, Annelise Tran, Laura Espinosa, Bertrand Sudre, Dragoslav Domanovic, and Shlomit Paz
Journal: Environmental Health
Reviewer: David Harley

General comments
Jan Semenza and the other authors of this paper present projections for West Nile Virus (WNV) in Europe. The findings of the paper are used to infer risk of transfusion-related transmission of WNV. The authors have used sophisticated methods incorporating climatic, epidemiological and biological data.

The paper is well written. Generally the methods are described in sufficient detail, and where they are not readers are referred to previous publications.

My two major criticisms are that the introduction requires attention as some statements are incorrect or difficult to defend, and that the authors overstate the usefulness of their modeling approach for projecting district level risk for WNV and the consequent threat to safe blood supply.

Major criticisms
The first sentence of the introduction is almost certainly untrue. Can the authors defend the assertion that WNV is "responsible for the largest outbreaks of fatal neuroinvasive disease in the world"? What infections are considered as causes of "fatal neuroinvasive disease"? Surely cerebral malaria, rabies, tuberculous meningitis, and opportunistic cerebral infections related to HIV are more important than WNV? Later in the introduction the authors state that "[g]lobally, WNV is the most widespread arthropod-borne virus…" Surely dengue is more widespread? But if the authors disagree they need to defend this statement. What do they mean by "widespread", exactly? The introduction is quite long and it is not entirely clear what the direct relevance for the work presented actually is. In the Introduction the paragraph beginning "[a]mbient temperature is another important environmental determinant…" is rather turgid and contains much that is fairly trite. The three significant points are 1/ WNV transmission is influenced by temperature because of effects on virus and vectors; 2/ field observations in Europe confirm this association; and 3/ parts of Europe will get hotter than the global average increase so there might be WNV hotspots in Europe. Surely these points can be made in fewer than 227 words?

The authors describe the sensitivity of their model for detecting districts with outbreaks as "moderate". Given that the sensitivity is only 0.24 this seems a bold and arguably misleading statement, as slightly less than a quarter of districts with outbreaks would be detected using the authors' approach. This is a very significant issue for the paper and the authors need to give much greater consideration to the implications, including for the significance of the research they have reported.

Minor criticisms
In the first paragraph of the abstract the meaning of the phrase "effective determinants" is unclear. Reading the final sentence of the first paragraph of the abstract I was left wondering what "intrinsic factors of the vector and virus" are considered to be important. The authors should be a little more specific regarding the virus characteristics that are particularly important as "effective determinants of WNV outbreaks". Presumably the authors are alluding to virus characteristics influencing virulence in humans and intrinsic and extrinsic incubation periods, and vector characteristics that present barriers to transmission ecologically (e.g. abundant non-human blood sources, localized behavioural variation in blood meal preference) or biologically (e.g. midgut infection and escape barriers). In the introduction, the sentence "[t]wo types of land-use have been associated with outbreaks in Europe, namely rural and urban areas" is rather labored and clumsy. Why not just write, "European outbreaks of WNV occur in rural and urban areas"?

The A1B emissions scenario is used, the authors write, because it is "balanced". This seems like jargon. A less glib explanation needs to be given to justify the use of this scenario.

In Table 1 an exposure variable called "Migration" takes values of either "Western path" or "Eastern path". The meaning of these terms is not made clear. Please explain the terms and justify the significance for the reader.

Reviewer 2

Review

Climate Change Projections of West Nile Virus Infections in Europe: Implications for Blood Safety Practices

The authors present an interesting article about the potential expansion of West Nile Virus (WNV) infections in Europe with climate change. They also address an important public health issue regarding the transmission of WNV via blood transfusion. The manuscript is generally clear and well written. The following questions and suggestions could help to strengthen the manuscript:

Abstract

Briefly mention what A1B scenario is.
Change “will also expand in the future” to “is expected to expand in the future”

Introduction

“experience only with flu-like symptoms” - remove word “with”

“and Ukraine, but since 1996 has caused...” → change to “and Ukraine. However, since 1996 WNV has caused...”

Before the discussion about outbreaks in rural and urban areas, it would be useful to briefly explain the transmission cycle and different hosts (e.g. birds, horses, humans...)

“Two types of land-use have been associated with outbreaks in Europe, namely rural and urban areas” → change to “WNV outbreaks occurs in both rural and urban areas”

Last paragraph of introduction:
-explain that the different predictors were tested in a statistical model.
-explain what the A1B climate change scenario is here, and place it in context with the range of different scenarios available.

Methods

Change sub title to “Temperature data”

Are the monthly anomalies for mean temperature? (or max or min?)

When describing WNV epidemiology, remind the reader when the WNV transmission season in Europe takes place.

Is the sentence about temperature data at the end of the WNV epi section needed?

Environmental variables: explain the motivation for using NDVI/MNDWI values. How could they help to predict WNV transmission?

Multivariate models: Although the reference to the article describing the model is included, it would be useful to include here some more details about the model, e.g. the probability distribution assumed (Gaussian? Binomial) and how the response variable was constructed (e.g. frequency?)
“the occurrence of a WND outbreak of the previous year”
On page 7, should WND read WNV? Change “of” to “in”

“logistic regression models’ coefficients” → change to “parameter estimates”

“λ: Weighted average of the number of infected districts amongst the neighbourhood the previous year” - Does the neighbourhood refer to the neighbouring NUTS3 regions?

“July temperature anomalies were entered for each year between 2015 and 2050 in order to compute the probability of WNV infections per district and per year which is one of the parameters in the model (infection the previous year)[10].” - Is this parameter λ? If so, include in text.

Provide some more details about the EUFRAT tool.

Results

How was a “correct prediction” defined? Did you use a threshold probability to decide if the model correctly predicted an outbreak or not?

Conclusion

“Areas at risk for current..” → “Areas at risk of current....”

Figures

In Fig 6 it is not clear what part A and B are showing. It would be good to explain in the caption. The authors might consider using a different colour scale to show old and new districts (so as not to confuse with the probability maps).

In Fig 7 it would be good to explain the difference between the prevalence estimates and the probability maps, and if relevant, consider using a different colour to show prevalence.

Reviewer 3

Climate Change Projections of West Nile Virus Infections in Europe:

Implications for Blood Safety Practices

Jan C. Semenza[1*], Annelise Tran[2], Laura Espinosa[1], Bertrand Sudre[1],

Dragoslav Domanovic[1], Shlomit Paz[3]

1: European Centre for Disease Prevention and Control, Stockholm, Sweden
2: CIRAD, UPR Animal et Gestion Intégrée des Risques, Montpellier, France
Montpellier, France
3: Department of Geography and Environmental Studies, University of Haifa, Mt. Carmel, Haifa, Israel

*Contact information for corresponding author:
Professor
Jan C. Semenza
European Centre for Disease Prevention and Control
SE-171 83 Stockholm
Sweden
Fixed Line: +46-(0)8-5860-1217
Cell: +46-(0)76-101-0711
Fax: +46-(0)8-5860-1296
Email: Jan.Semenza@ecdc.europa.eu

Running title: Projections of West Nile Virus Infections

Key Words: West Nile fever, West Nile virus, climate change, blood safety, blood supply, environmental determinants, epidemiology, temperature, surveillance, arbovirus, remote sensing, risk maps

Abstract

Background:

West Nile virus (WNV) is transmitted by mosquitoes in both urban as well as in rural environments and can be pathogenic in birds, horses and humans. Extrinsic factors such as temperature and land use are effective determinants of WNV outbreaks in Europe, along with intrinsic factors of the vector and virus.

Methods:

With a multivariate model for WNV transmission we computed the probability of WNV infection in 2014, with July 2014 temperature anomalies. We applied the July temperature anomalies under the A1B climate change scenario for 2025 and 2050 to model and project the risk of WNV infection in the future. Since asymptomatic infections are common in humans (which can result in the contamination of the donated blood) we estimated the predictive prevalence of WNV infections in the blood donor population.

Results:

External validation of the probability model with 2014 cases indicated good prediction, based on an Area Under Curve (AUC) of 0.871 (SD=0.032), on the Receiver Operator Characteristic Curve (ROC). The climate change projections for 2025 reveal a higher probability of WNV infection particularly at the edges of the current transmission areas (for example in Eastern Croatia, NorthEastern Greece and Northwestern Turkey) and an even further expansion in 2050. The prevalence of infection in (blood donor) populations in the outbreak-affected districts will also expand in the future.

Discussion:

Predictive modelling of environmental and climatic drivers of WNV can be a valuable tool for public health practice. It can help delineate districts at risk for future transmission. These areas can be subjected to integrated disease and vector surveillance, outreach to the public and health care providers, implementation of personal protective measures, screening of blood donors, and vector abatement activities.

Introduction

West Nile virus (WNV) is responsible for the largest outbreaks of fatal neuroinvasive disease in the world [1]. WNV infections occur predominantly through mosquito bites but also through blood transfusion or organ, tissue and cell-transplantations. Most human infections are asymptomatic and mild cases experienced only with flu-like symptoms; more severe cases present with signs of encephalitis, meningo-encephalitis or meningitis. Globally, WNV is the most widespread arthropod-borne virus, an enveloped, single-strand RNA virus of the genus *Flavivirus* in the family of *Flaviviridae* [2-4]. It circulates in Africa, Americas, Asia, Europe, and Australia, where it is thought have been introduced from the Middle East [4]. In Europe, Middle East and Africa, WNV has been responsible for sporadic outbreaks in the 1950s in Israel, in the 1960s in Russia and France, in the 1970s in Belarus, South Africa, and Ukraine, but since 1996 has caused more recurrent outbreaks in Europe and northern Africa [5-8]. In 2010, large outbreaks in humans occurred in Southeastern and Eastern Europe. The European outbreaks occurred in Russia, the Czech Republic, Hungary, Romania, Turkey, Greece, Italy, France, Spain and Portugal [9]. Since 2010, there have been annual outbreaks in Southeastern and Eastern Europe, suggesting an endemic transmission cycle and thus a resurgent public health problem [10]. Two types of land-use have been associated with outbreaks in Europe, namely rural and urban areas. They respectively (and independently) contribute to high concentrations of hosts with competent mosquito vectors that support intense local avian transmission. Rural areas with estuaries, wetlands or marshes attract migratory birds for breeding, nesting, and rearing their young. These bird habitats also attract bird-feeding mosquitoes where congregating bird populations can get infected with WNV. In fact, a number of human outbreaks have originated in European estuaries such as in the Danube delta in Romania, in the Volga delta in Russia, and in the Rhone delta in France [11]. Urbanized areas can also attract large bird and mosquito populations where humans can get exposed [12] and local environmental conditions can increase the potential for mosquito breeding in urban settings [13]. For example, in 1996, a large outbreak occurred in the city of Bucharest, Romania that affected 4% of the population [7]. Over 800 patients were hospitalized during another large WNV outbreak that occurred in 1999 in Volgograd City on the west bank of the Great Volga River [14] and in 2010 WNV infections were documented for the first time in humans in the Greek city of Thessaloniki [15,16].

Certain metropolitan areas can sustain a high breeding density of birds; for example, European Starlings thrive on urban lawns or parks where they can feed, or gulls prolife[illegible]e near open water [17]. Birds from these types of urban environments harbour viruses with higher genetic diversity than birds from residential areas, indicating that anthropogenic factors associated with urbanization play an important role in arboviral transmission and evolution [18]. In the United States, WN[illegible]fection rates in crows and humans are higher in more urbanized environments that are less forested [19,20], while in Europe, WNV transmission can occur both in rural and urban areas.

Ambient temperature is another important environmental determinant in the transmission of WNV as it has a direct impact on mosquito survival, developmental rates of immature stages, growth rates of vector populations and decrease in the interval between blood meals [21-24]. Moreover, temperature also affects the extrinsic incubation period (the number of days from ingestion to transmission) by influencing the viral replication rates and thus the transmission of WNV [25,26]. In a modelling study elevated air temperature was the strongest predictor of increased infection in mosquito vectors [24]. As for Europe, it was found that the unprecedented upsurge in the number of human WNF cases in summer 2010 was accompanied by extremely hot spells in the region [21]. Moreover, recent research analysed the status of infection by WNV in Europe and its neighbouring countries in relation to environmental and climatic risk parameters. The anomalies of temperature in July were identified as one of the main risk factors [10]. During recent decades, parts of Europe have warmed up more than the global average. Additionally, more frequent and more intense hot extremes have occurred. This trend is expected to continue while predictions suggest a further temperature increase (between 1.0°C and 5.5°C) by the end of the century [27]. Increases in ambient temperature due to climate change are therefore projected to impact WNV transmission in Europe and its neighbouring countries [28-32].

In order to examine these land-use and climatic variables as predictors of the probability of WNV infection [4] we tested the contribution of remotely sensed temperature, the state of vegetation and water bodies, and bird migratory routes. We also project the WNV risk in Europe into 2025 and 2050, with July temperature projections under the A1B climate change scenario. Insights from these analyses can also be used to assess the current and future WNV risk to the safety of blood supply in the region [33]. Thus, we provide a

quantification of the risk of WNV transmission through blood transfusion by estimating the prevalence of infection in (donor) populations of an area affected by a WNV outbreak, as well as the infection risk of a blood donor that visited an outbreak affected area. In the long-run, the environmental determinants identified in this model lend themselves for an integration of environmental monitoring in public health surveillance systems of human cases, serological surveillance of domestic and wild avifauna, and entomological surveillance [4,34,35].

Methods

Temperature

Monthly anomalies of July 2014 temperature at the locations of WNF outbreaks reported in humans were computed and predicted surface temperatures and anomalies were extracted for 2015-2050 from NCAR climate change scenarios for the gridded region 30°N-60°N and 10°W-55°E [36]. The A1B Scenario was chosen since it is a balanced scenario; its main characteristics include: low population growth, very high GDP growth, very high energy use, low-medium land use changes, medium resource (mainly oil and gas) availability, rapid pace and direction of technological change favouring balanced development. The chosen model output is the Ensemble Average which is the mean state of the climate among all model runs. Global climate simulations were produced at NCAR by the Community Climate System Model (CCSM3) for the 4th Assessment report of the IPCC [37].

Climate simulations from the CCSM3 are generated on a Gaussian grid, where each grid point can be uniquely accessed by one-dimensional latitude and longitude arrays (i.e. the coordinates are orthogonal). In the CCSM3 model output, the longitudes are equally spaced at 1.40625º, while the latitudes vary in spacing from 1.389º to 1.400767º. Therefore, the approximate spatial resolution of the global climate projections is 155 km. Because of the irregular grid in the CCSM model, this portal distributes data in a point shape file format, where each point represents a centroid of a corresponding CCSM grid cell. Anomalies are relative to the 20th Century Experiment 1980-1999, based on NCAR methodology [37].

WNV epidemiology

The methods for the WNV epidemiological model in Europe have been described previously [10]. Briefly, the epidemiologic data of human West Nile cases were obtained from the West Nile fever surveillance conducted at ECDC during the transmission season in Europe [38]. Population data for the European Union were based on the nomenclature of territorial units for statistics classification (NUTS) at level 3 with population estimate of 2010 [39]. Global Administrative Unit Layers (GAUL) were used for regions outside of the European Union, and project and population estimates for the year 2010 were derived from the Gridded Population of the World (GPW) dataset [40]. The gridded data of monthly mean of air temperature for the region between 30°N-60°N and 10°W-55°E was obtained from the NOAA NCEP-NCAR database [41].

Environmental variables

To derive the Normalized difference vegetation index (N[illegible]) and Modified normalized difference water index (MNDWI) values, Moderate resolution imaging spectroradiometer (MODIS) data products were acquired from Land Process Distributed Active Archive Center (LP DAAC). MODIS Terra 8-day composite images of surface reflectance estimates at 500 m spatial resolution (product MOD09A1) were acquired for all WNV infected countries for a twelve years period (2002–2013). The long-term average and standard deviation of each of the environmental indices were computed on monthly bases for the temperature data, and on 8-days interval bases for the MODIS NDVI and MNDWI data. The anomaly (z) of temperatures, NDVI and MNDWI was calculated for each date i (month or 8-days period) as a function of the annual indices x_i and their long-term average and standard deviation values. The mean anomalies of temperatures, NDVI and MNDWI were computed for each district and each month and MODIS 8-days period. The analysis was performed at the district level (n=1113) categorized as ‘infected’ if WNV human cases were reported there that year, and as ‘non-infected’ otherwise.

Multivariate models

We used multivariate logistic regression models to test the probability of an ‘infected district’ as the response variable, and as explanatory variables the population, the presence

of wetlands, the presence of birds' migratory routes, the anomalies of temperature, NDVI and MNDWI [10]. We also tested as explanatory variable: the occurrence of a WND outbreak of the previous year, considering that WNV could persist locally through survival in overwintering mosquitoes or infected birds. A bootstrap procedure (1,000 replicates) was applied to estimate the 95% confidence interval (95% CI) of the logistic regression models' coefficients, selecting randomly each time from the original set of 1113 districts 90% of infected districts between 2002 and 2011 (n = 98) and 90% of non-infected districts (n = 903). The final model used in the current analysis is described in Table 1. This model was validated using 2012-2013 epidemiologic data not used for model construction [10] :

Table 1. Multivariate logistic regression model parameter of the risk of WNV infection at district level, EU and neighbouring countries [10].

	Parameter	95% CI	p-value
Intercept	-5.85	[-6.02;-5.74]	-
TMPJUL	**0.37**	[0.32;0.41]	<10-7
MNDWI21	**1.14**	[1.06;1.22]	<10-15
λ	**5.06**	[4.78;5.31]	<10-15
WETLANDS<br>Absence<br>Presence	 <br>**1.38**	 <br>[1.16;1.55]	 <br><10-7
MIGRATION<br>Western path<br>Eastern path	 <br>**1.04**	 <br>[0.91;1.24]	 <br><10-7
POPULATION	1.66 10-7	[1.66 10-7;2.21 10-7]	<10-2

Significant variables are highlighted in bold characters

TMPJUL: Monthly anomalies for July temperature from the perennial mean monthly temperature

MNDWI21: 8 days anomalies for June Modified Normalized Difference Water Index

λ: Weighted average of the number of infected districts amongst the neighbourhood the previous year

The average, standard deviation, and anomalies of the 2014 temperature was computed and applied to the model described above [10]. The results were compared with the actual occurrence of WNV in 2014. Validity of the model was assessed based on the ability of the model to distinguish between districts with and without WNV, using sensitivity, specificity, and Area Under Curve (AUC) of the Receiver Operator Characteristic Curve (ROC).

Climate change projections

The model was used to predict the probability of WNV infection by applying the projected July temperatures for 2025 and 2050. We extracted gridded temperature projections for the A1B Scenario, which is a balanced climate change scenario [37]. July temperature anomalies were entered for each year between 2015 and 2050 in order to compute the probability of WNV infections per district and per year which is one of the parameters in the model (infection the previous year)[10].

Projections of the prevalence of infection

To assess the safety of the blood supply from a WNV infection we used the European Up-Front Risk Assessment Tool (EUFRAT) developed by ECDC [42]. We calculated the prevalence of infection in (donor) population in affected areas and the probability of the blood donor to become infected after visiting such an area. For the calculation, the absolute number of donors infected with WNV in the outbreak-affected area was used from the notification/surveillance systems reported to ECDC. This number refers to individuals in the population of the outbreak-affected areas that are reported/ notified as cases when they seek health care during the epidemic. We used 60% as a conservative estimate of the proportion of undetected cases which includes WNV infected individuals that may not develop symptoms, did not seek for health care, or were misdiagnosed with other diseases and were thus not reported as cases. The population size was entered as the population number in the outbreak-affected areas where the notified cases were recorded. The proportion of chronic infection was set to zero. The duration of the epidemic was the length of period since the first case was reported up until the last day of reported cases and was set to 300 days. The prevalence of infection in the (donor) population was calculated based on these parameters for 2014.

Results

Temperature anomalies in Europe for July 2014 are shown in figure 1. The locations of 210 probable and confirmed cases of WNV infections in Europe and neighbouring countries for 2014 are illustrated in figure 2. An affected area was defined as an area with one or more autochthonous human WNV cases which were recorded in a number of countries: Austria (1); Greece (15); Hungary (11); Italy (24); Romania (23); Bosnia and Herzegovina (13); Israel (17); Palestine (1); Russian Federation (29); and Serbia (76). We used the multivariate logistic regression model developed by Tran et al to compute the probability of WNV occurrence based on July temperatures anomalies, the anomaly of the Modified Normalized Difference Water Index (MNDWI) in early June, an outbreak of the previous year, the size of the human population, wetlands [illegible] the type of avian flyways [10]. The probability map with the July 2014 temperature deviation is presented in figure 3. Geographic areas of predicted probability of high WNV infection were North-eastern Greece, central Hungary, North-eastern Italy, Eastern Romania, central Serbia, and large areas of Southern Russia. This probability map was compared with the actual occurrence of WNV cases in 2014 (figure 2) and a concordance was observed. Validity of the model was assessed based on the ability of the model to distinguish between districts with and without WNV. External validation of the model with the 2014 data indicated good prediction based on the Receiver Operator Characteristic Curve (ROC) (figure 4). The Area Under Curve (AUC) of the model reached 0.871 (SD=0.032). Sensitivity was calculated as the proportion of WNV positive districts correctly identified by the model and yielded 0.236 (SD=0.074). Specificity was calculated as the proportion of WNV negative districts correctly identified by the model and yielded 0.982 (SD=0.006).

Probability maps were generated with the A1B temperature projections (figure 5). The results reveal a progressive expansion of areas with an elevated probability for WNV infections, particularly at the edges of the transmission areas. For example, the 2025 map reveals a higher probability of WNV infection in Eastern Croatia, NorthEastern Greece and Northwestern Turkey. In 2050, the area with a higher probability will have expanded even more with a total of 147 and 405 districts being affected in 2025 and 2050, respectively. A total of 81 and 268 new districts recorded WNV infections for the first time in 2025 and

2050 compared to 2014 that were situated on the perimeter of the transmission areas (figure 6).

WNV infections are a significant concern to the safety of the blood supply, because the blood donated by asymptomatic carriers might inadvertently contaminate the blood supply [33]. Therefore, we calculated the prevalence of infection in (donor) populations in the outbreak-affected areas. We mapped the donor population infectivity (figure 7) and found an extended area of elevated WNV infection hazard for the safety of the blood supply in 2025 compared to 2014.

Discussion

Projections of WNV risk

The multivariate model for WNV outbreaks developed for the period 2002 to 2011 and validated with 2012 and 2013 data [10] was applied here to July 2014 temperature anomalies. The probability map revealed a good agreement with the actual WNV outbreaks in 2014, based on the ROC curve. The ability of the model to detect a district with WNV infections was moderate (sensitivity = 0.236) while the ability of the model to correctly detect a negative district was very good (specificity = 0.982). These findings indicate that July temperature anomalies can be used in our prediction model as an early warning of the coming WNV season and help delineate areas of imminent transmission. These areas can then be targeted for enhanced epidemiologic surveillance of neuro-invasive illness (which may be suggestive of WNV infection), awareness rising among healthcare workers for the clinical presentation of WNV infection, and reinforced laboratory diagnostic capacity. Moreover, these areas can be subjected to entomological surveillance to characterize mosquito breeding sites, and vector abatement measures to diminish mosquito densities. Passive surveillance in domestic birds and equine populations to monitor the dispersion of WNV might also be considered.

This model was also applied to July temperature projections for 2025 and 2050 in order to quantify the future burden of WNV. These results indicate that 81 and 268 districts will be impacted by WNV outbreaks by 2025 and 2050, respectively. Districts adjacent to districts with current transmission are at elevated risk and should therefore also be considered for the public health interventions listed above.

Limitations

The projections presented in this paper are computed on the assumption that the other variables remained stable. For example, the population size was maintained constant in these projections; however the contribution of the population size to the model output is very minimal, based on a low parameter value (1.66 10^{-7}). Moreover, the Easter bird migration path was retained in the model, as well as the presence of wetlands, and the MNDWI21 under the assumption that they will not change significantly. Yet, other WNV determinants that were not part of our model could change over the given time period. For example, mosquito control measures could differ at the area and country level and change over time, with implications for WNV incidence. Similarly, the quality of surveillance clinical case detection might differ too and change over time. WNV might spread at different rates in local, amplifying bird populations that might eventually develop herd immunity. Moreover, other environmental determinates might vary over time as well. Thus, although there are a number of limitations to our model, its validation performed rather well based on the sensitivity and specificity calculations for WNV infections in 2012, 2013 [10] and 2014.

Blood supply safety

The arrival and dispersal of tropical pathogens to Europe and its neighbouring countries commonly associated with warmer temperatures pose a threat to the supply of safe blood products, particularly if they are unknown or without diagnostic tests [33,43]. Thus, emerging infectious diseases will continue to pose a threat to transfusion safety on European and international levels [44-46]. Specifically, a progressive expansion of areas with an elevated probability for WNV infections will increase the threat to the safety of blood transfusion. In geographically larger areas affected by WNV, a higher number of blood donors will be exposed to infection for a longer time period (if the duration of the annual mosquito activity season will be prolonged). Our climate change projections of the predicted probability of WNV infection in Europe have far reaching implications for public health in the future, because the findings in this paper can contribute to WNV preparedness activities. Besides the cases of primary WNV infections, secondary infections through contaminated blood products are of increasing concern to threaten the safety of the blood

supply [33]. The asymptomatic blood-borne phase of a WNV infection increases the potential for transmission by transfusion, even if it is relatively short, compared to Hepatitis B virus or HIV [47]. Moreover, WNV has the ability to survive and persist in collected blood and stored blood components and subsequently cause an infection through the intravenous application. Thus, we calculated the prevalence of infection in the donor populations in the outbreak-affected areas (Figure 7). However, this instantaneous estimate may underestimate the true prevalence of infection if the timing of the WNV epidemic is at the peak of the epidemic curve. Nevertheless, the map reveals considerable vulnerabilities in Southeastern Europe when it comes to the safety of the blood supply. These insights can help transfusion service and clinical staff identify, manage and plan for transfusion-transmitted infections. The overall management of blood safety should be addressed at the institutional level, specifically at regulatory agencies or professional organizations [47]. To preserve the number of eligible donors and an adequate blood supply, authorities will inevitably reassess risk reduction interventions [33]. These include deferral strategies [48,49], screening strategies and triggers [50-52], and also pathogen reduction technologies [33,53-55]. Moreover, it might be necessary to distribute blood components to outbreak areas from unaffected areas in order to prevent intermittent shortfalls in the blood supply [56]. Such coordination requires supra-national inventories of blood products that can be dispatched upon demand. Therefore, the projected temperature change with elevated probability for WNV infections and possible increased prevalence of WNV infected blood donors should be taken into account in developing preparedness plans for the WNV safety of the blood supply. This could substantially increase the costs of blood transfusion therapy. The projections presented here may therefore offer an insight into future developments in the risk of transfusion from WNV infections; it also provides an opportunity to timely define the optimal strategy of blood safety in the face of limited available resources.

Conclusion

In several countries of Southeastern Europe, WNV transmission is now established. Our predictive model suggests further WNV dispersal in the coming years to adjacent districts. Monitoring and modelling climatic and environmental conditions permissive for the interaction of migratory birds, resident birds, competent mosquito vectors and humans can help delineate districts at risk of transmission. Areas at risk for current and future WNV

transmission can be targeted for integrated surveillance, vector control measures, outreach to the public and health care sector, strengthened laboratory capacity for reliable WNV diagnosis, and systematic screening of blood donors [57]. These activities call for inter-sectorial collaboration to tackle the challenges of WNV transmission.

Acknowledgement

The authors would like to thank Mikel Santamaria Guerro for his assistance with the maps and Drs. Yvan Hutin and Mike Catchpole at ECDC for critical feedback on the manuscript. The study was funded by ECDC and several co-authors (JCS, LE, BS, DD) are employed by ECDC. The authors declare no financial competing interests. The opinions in this paper reflect that of the authors and not necessarily that of ECDC.

References

1. Petersen LR, Brault AC, Nasci RS (2013) West Nile virus: review of the literature. JAMA 310: 308-315.
2. Ciota AT, Kramer LD (2013) Vector-virus interactions and transmission dynamics of West Nile virus. Viruses 5: 3021-3047.
3. May FJ, Davis CT, Tesh RB, Barrett AD (2011) Phylogeography of West Nile virus: from the cradle of evolution in Africa to Eurasia, Australia, and the Americas. J Virol 85: 2964-2974.
4. Ozdenerol E, Taff GN, Akkus C (2013) Exploring the spatio-temporal dynamics of reservoir hosts, vectors, and human hosts of West Nile virus: a review of the recent literature. Int J Environ Res Public Health 10: 5399-5432.
5. Hayes C (1989) West Nile Fever. In: Monath TP, editor. The Arboviruses: Epidemiology and Ecology. Boca Raton, FL, USA: CRC Press.
6. Karabatsos N (1985) International Catalogue of Arbovirus Including Certain Other Viruses of Vertebrate. San Antonio, TX, USA: American Society of Tropical Medicine and Hygiene.
7. Tsai TF, Popovici F, Cernescu C, Campbell GL, Nedelcu NI (1998) West Nile encephalitis epidemic in southeastern Romania. Lancet 352: 767-771.
8. Lvov DK, Butenko AM, Gromashevsky VL, Larichev VP, Gaidamovich SY, Vyshemirsky OI, Zhukov AN, Lazorenko VV, Salko VN, Kovtunov AI, Galimzyanov KM, Platonov AE, Morozova TN, Khutoretskaya NV, Shishkina EO, Skvortsova TM (2000) Isolation of two strains of West Nile virus during an outbreak in southern Russia, 1999. Emerg Infect Dis 6: 373-376.
9. Paz S, Semenza JC (2013) Environmental drivers of West Nile fever epidemiology in Europe and Western Asia--a review. Int J Environ Res Public Health 10: 3543-3562.
10. Tran A, Sudre B, Paz S, Rossi M, Desbrosse A, Chevalier V, Semenza JC (2014) Environmental predictors of West Nile fever risk in Europe. Int J Health Geogr 13: 26.
11. Hubalek Z, Halouzka J (1999) West Nile fever--a reemerging mosquito-borne viral disease in Europe. Emerg Infect Dis 5: 643-650.
12. Deichmeister JM, Telang A (2011) Abundance of West Nile virus mosquito vectors in relation to climate and landscape variables. J Vector Ecol 36: 75-85.
13. Epstein PR (2001) West Nile virus and the climate. J Urban Health 78: 367-371.
14. Platonov AE, Shipulin GA, Shipulina OY, Tyutyunnik EN, Frolochkina TI, Lanciotti RS, Yazyshina S, Platonova OV, Obukhov IL, Zhukov AN, Vengerov YY, Pokrovskii VI (2001) Outbreak of West Nile virus infection, Volgograd Region, Russia, 1999. Emerg Infect Dis 7: 128-132.
15. Papa A, Danis K, Baka A, Bakas A, Dougas G, Lytras T, Theocharopoulos G, Chrysagis D, Vassiliadou E, Kamaria F, Liona A, Mellou K, Saroglou G, Panagiotopoulos T (2010) Ongoing outbreak of West Nile virus infections in humans in Greece, July-August 2010. Euro Surveill 15.
16. Danis K, Papa A, Theocharopoulos G, Dougas G, Athanasiou M, Detsis M, Baka A, Lytras T, Mellou K, Bonovas S, Panagiotopoulos T (2011) Outbreak of West Nile virus infection in Greece, 2010. Emerg Infect Dis 17: 1868-1872.
17. Savard JL, Clergeau P, Mennechez G (2000) Biodiversity concepts and urban ecosystems. Landsc Urban Plan 48: 131-142.
18. Bertolotti L, Kitron UD, Walker ED, Ruiz MO, Brawn JD, Loss SR, Hamer GL, Goldberg TL (2008) Fine-scale genetic variation and evolution of West Nile Virus in a transmission "hot spot" in suburban Chicago, USA. Virology 374: 381-389.
19. LaDeau SL, Calder CA, Doran PJ, Marra PP (2011) West Nile Virus impacts in american crow populations are associated with human land use and climate. Ecol Res 26: 909-916.
20. Brown H, Duik-Wasser M, Andreadis T, Fish D (2008) Remotely-sensed vegetation indices identify mosquito clusters of West Nile virus vectors in an urban landscape in the northeastern United States. Vector Borne Zoonotic Dis 8: 197-206.

21. Paz S, Malkinson D, Green MS, Tsioni G, Papa A, Danis K, Sirbu A, Ceianu C, Katalin K, Ferenczi E, Zeller H, Semenza JC (2013) Permissive summer temperatures of the 2010 European West Nile fever upsurge. PLoS One 8: e56398.
22. Paz S, Albersheim I (2008) Influence of warming tendency on Culex pipiens population abundance and on the probability of West Nile fever outbreaks (Israeli Case Study: 2001-2005). Ecohealth 5: 40-48.
23. Meyer RP, Hardy JL, Reisen WK (1990) Diel changes in adult mosquito microhabitat temperatures and their relationship to the extrinsic incubation of arboviruses in mosquitoes in Kern County, California. J Med Entomol 27: 607-614.
24. Ruiz MO, Chaves LF, Hamer GL, Sun T, Brown WM, Walker ED, Haramis L, Goldberg TL, Kitron UD (2010) Local impact of temperature and precipitation on West Nile virus infection in Culex species mosquitoes in northeast Illinois, USA. Parasit Vectors 3: 19.
25. Reisen WK, Fang Y, Martinez VM (2006) Effects of temperature on the transmission of west nile virus by Culex tarsalis (Diptera: Culicidae). J Med Entomol 43: 309-317.
26. Andrade CC, Maharaj PD, Reisen WK, Brault AC (2011) North American West Nile virus genotype isolates demonstrate differential replicative capacities in response to temperature. J Gen Virol 92: 2523-2533.
27. EEA (2012) Climate change, impacts and vulnerability in Europe 2012. An indicator-based report. No 12/2012.
28. Greer A, Ng V, Fisman D (2008) Climate change and infectious diseases in North America: the road ahead. CMAJ 178: 715-722.
29. Platonov AE, Fedorova MV, Karan LS, Shopenskaya TA, Platonova OV, Zhuravlev VI (2008) Epidemiology of West Nile infection in Volgograd, Russia, in relation to climate change and mosquito (Diptera: Culicidae) bionomics. Parasitol Res 103 Suppl 1: S45-53.
30. Morin CW, Comrie AC (2010) Modeled response of the West Nile virus vector Culex quinquefasciatus to changing climate using the dynamic mosquito simulation model. Int J Biometeorol 54: 517-529.
31. Morin CW, Comrie AC (2013) Regional and seasonal response of a West Nile virus vector to climate change. Proc Natl Acad Sci U S A 110: 15620-15625.
32. Paz S (2015) Climate change impacts on West Nile virus transmission in a global context. Philos Trans R Soc Lond B Biol Sci 370.
33. Semenza JC, Domanović D (2013) Blood supply under threat. Nature Climate Change 3: 432-435.
34. Kwan JL, Park BK, Carpenter TE, Ngo V, Civen R, Reisen WK (2012) Comparison of enzootic risk measures for predicting West Nile disease, Los Angeles, California, USA, 2004-2010. Emerg Infect Dis 18: 1298-1306.
35. Semenza J, Zeller H (2014) Integrated surveillance for prevention and control of emerging vector-borne diseases in Europe. Euro Surveill 19.
36. NCAR Climate change scenarios.
37. Pachauri RKaR, A. (Eds.) (2007) Contribution of Working Groups I, II and III to the Fourth Assessment Report of the Intergovernmental Panel on Climate Change.
38. European Center for Disese Prevention and Control (ECDC) (2014) West Nile fever maps.
39. EUROSTAT (2014) Nomenclature of territorial units for statistics.
40. NASA (2014) Socioeconomic Data and Application Centre (SEDAC): Gridded population of the world (gpw), v3.
41. Kalnay E, Kanamitsu M, Kistler R, Collins W, Deaven D, Gandin L (1996) The ncep/ncar 40-year reanalysis project. Bull Am Meteorol Soc 77: 437–471.
42. ECDC European Up-Front Risk Assessment Tool (EUFRAT) http://eufrattool.ecdc.europa.eu/.
43. Semenza JC, Sudre B, Miniota J, Rossi M, Hu W, Kossowsky D, Suk JE, Van Bortel W, Khan K (2014) International dispersal of dengue through air travel: importation risk for Europe. PLoS Negl Trop Dis 8: e3278.

44. Dodd RY, Leiby DA (2004) Emerging infectious threats to the blood supply. Annu Rev Med 55: 191-207.
45. Alter HJ, Stramer SL, Dodd RY (2007) Emerging infectious diseases that threaten the blood supply. Semin Hematol 44: 32-41.
46. Stramer SL (2014) Current perspectives in transfusion-transmitted infectious diseases: emerging and re-emerging infections. ISBT Sci Ser 9: 30-36.
47. Stramer SL, Hollinger FB, Katz LM, Kleinman S, Metzel PS, Gregory KR, Dodd RY (2009) Emerging infectious disease agents and their potential threat to transfusion safety. Transfusion 49 Suppl 2: 1S-29S.
48. Orton SL, Stramer SL, Dodd RY (2006) Self-reported symptoms associated with West Nile virus infection in RNA-positive blood donors. Transfusion 46: 272-277.
49. Lieshout-Krikke RW, Zaaijer HL, Prinsze FJ (2013) The yield of temporary exclusion of blood donors, exposed to emerging infections abroad. Vox Sang 104: 12-18.
50. Busch MP, Caglioti S, Robertson EF, McAuley JD, Tobler LH, Kamel H, Linnen JM, Shyamala V, Tomasulo P, Kleinman SH (2005) Screening the blood supply for West Nile virus RNA by nucleic acid amplification testing. N Engl J Med 353: 460-467.
51. Kleinman SH, Williams JD, Robertson G, Caglioti S, Williams RC, Spizman R, Morgan L, Tomasulo P, Busch MP (2009) West Nile virus testing experience in 2007: evaluation of different criteria for triggering individual-donation nucleic acid testing. Transfusion 49: 1160-1170.
52. Custer B, Tomasulo PA, Murphy EL, Caglioti S, Harpool D, McEvoy P, Busch MP (2004) Triggers for switching from minipool testing by nucleic acid technology to individual-donation nucleic acid testing for West Nile virus: analysis of 2003 data to inform 2004 decision making. Transfusion 44: 1547-1554.
53. Gallian P, Vignoli C, Dombey AM, Mayaudon V, Lin L, Galichet V, Cantaloube JF, De Micco P (2006) Inactivation of a European strain of West Nile virus in single- donor platelet concentrate using the INTERCEPT blood system. Vox Sang 91: 345-347.
54. Vanlandingham DL, Keil SD, Horne KM, Pyles R, Goodrich RP, Higgs S (2013) Photochemical inactivation of chikungunya virus in plasma and platelets using the Mirasol pathogen reduction technology system. Transfusion 53: 284-290.
55. Burnouf T, Chou ML, Cheng LH, Li ZR, Wu YW, El-Ekiaby M, Tsai KH (2013) Dengue virus inactivation by minipool TnBP/Triton X-45 treatment of plasma and cryoprecipitate. Vox Sang 104: 1-6.
56. Bambrick HJ, Woodruff RE, Hanigan IC (2009) Climate change could threaten blood supply by altering the distribution of vector-borne disease: an Australian case-study. Glob Health Action 2.
57. Semenza JC, Zeller H (2014) Integrated surveillance for prevention and control of emerging vector-borne diseases in Europe. Euro Surveill 19.

Figure 1. Temperature anomalies for July 2014

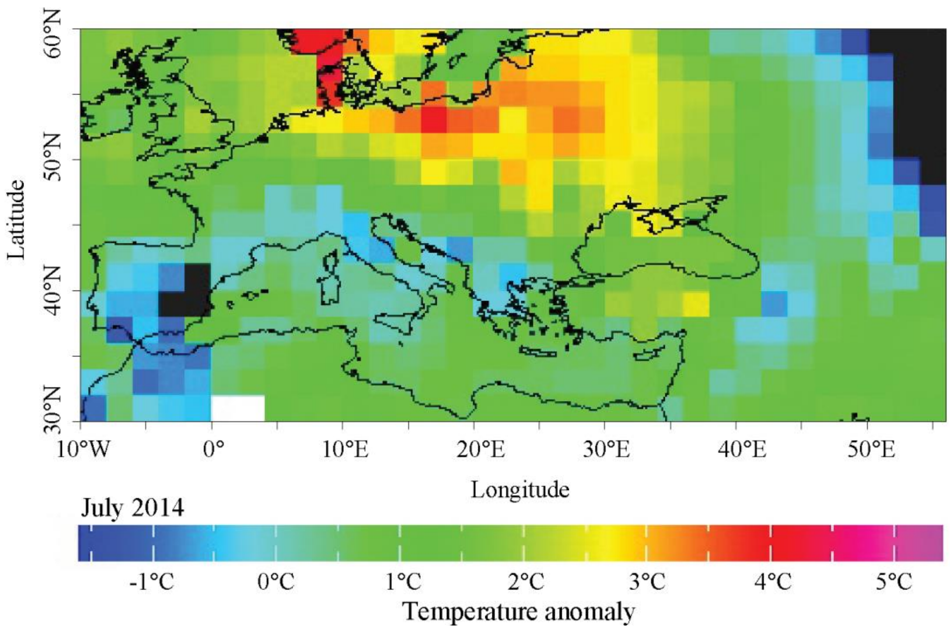

Figure 2. Districts with probable and confirmed cases of West Nile Virus infections, as of 20/11/2014.

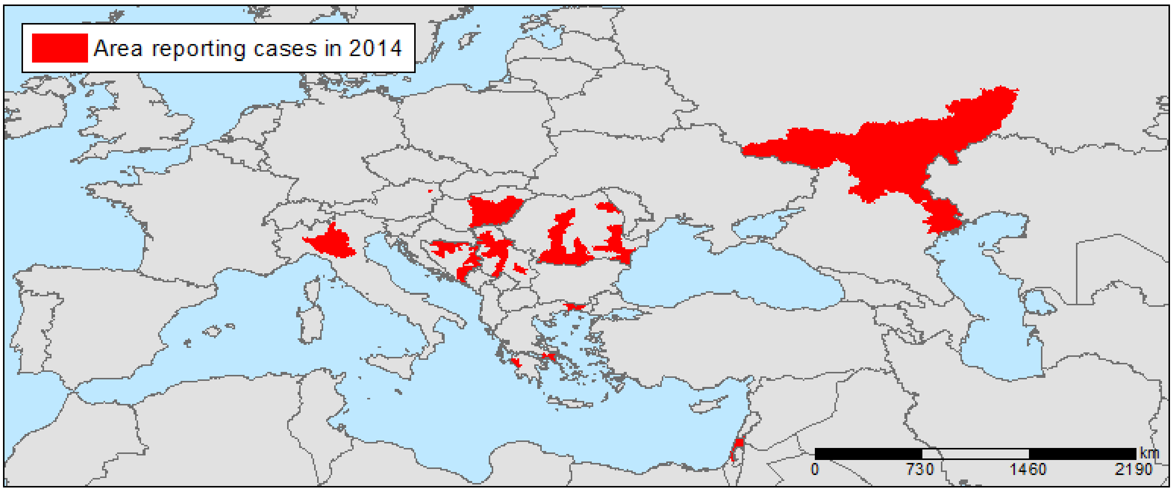

Note: An affected area is defined as an area with one or more autochthonous human WNV cases (neuro-invasive and non neuro-invasive), meeting laboratory criteria as per EU case definition' (Directive 2008/426/EC). WNV cases by country: Austria (1); Greece (15); Hungary (11); Italy (24); Romania (23); Bosnia and Herzegovina (13); Israel (17); Palestine (1); Russian Federation (29); and Serbia (76).

Figure 3. Predicted probability of districts with West Nile Virus infections for 2014.

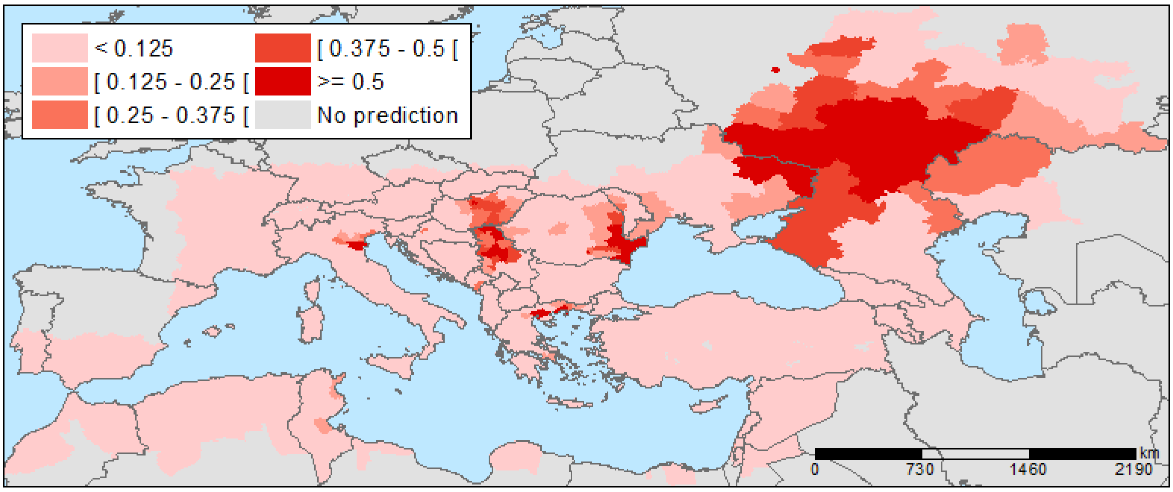

Figure 4. Receiver operator characteristic curve of the probability of West Nile Virus infections in 2014.

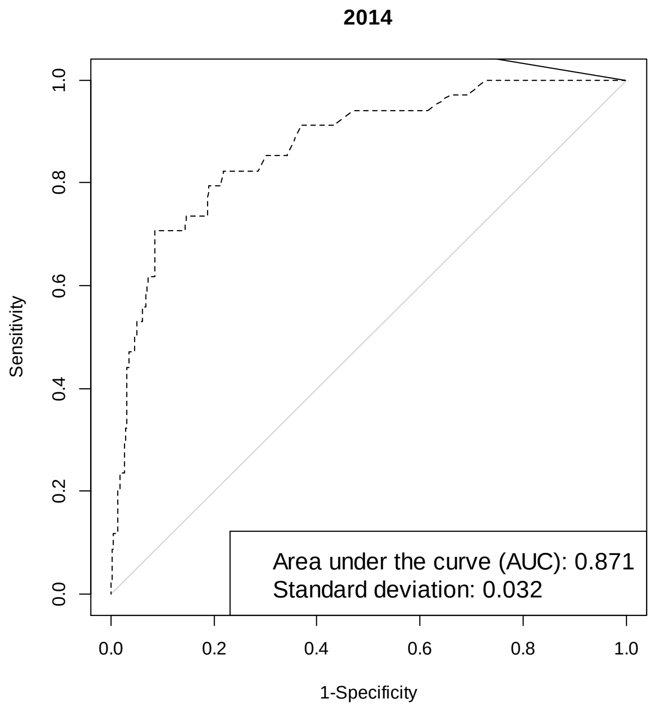

Figure 5. Predicted probability of districts with West Nile Virus infections based on July temperatures for A1B scenario projections for 2025 (A) and 2050 (B).

A.

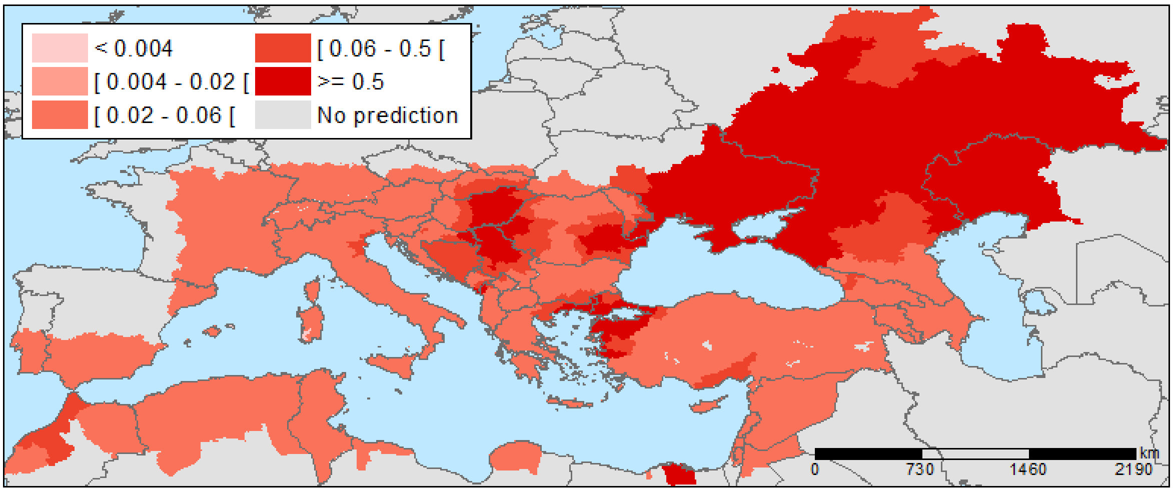

B.

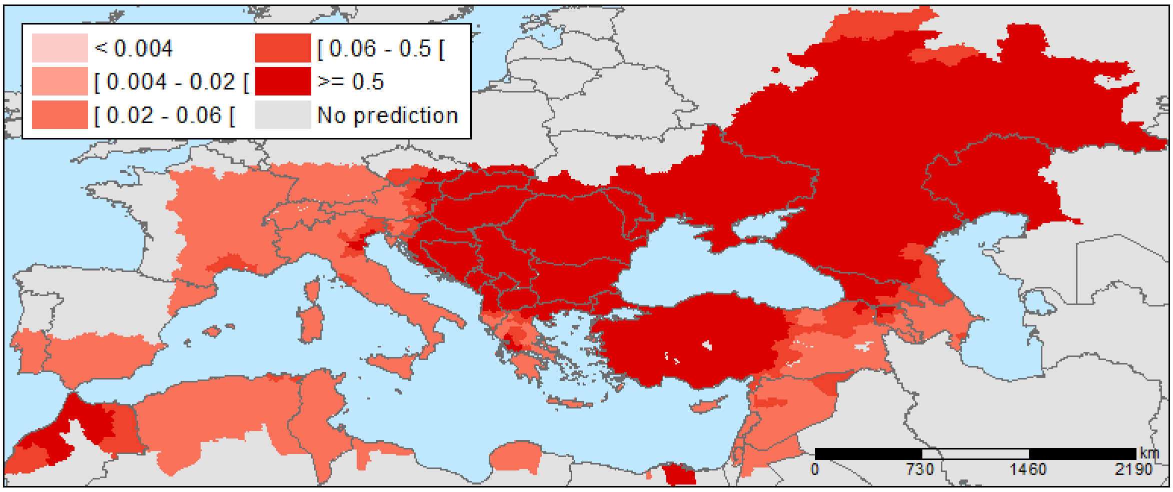

Figure 6. New districts affected by West Nile Virus infections in 2025 compared to 2014.

A. Confirmed

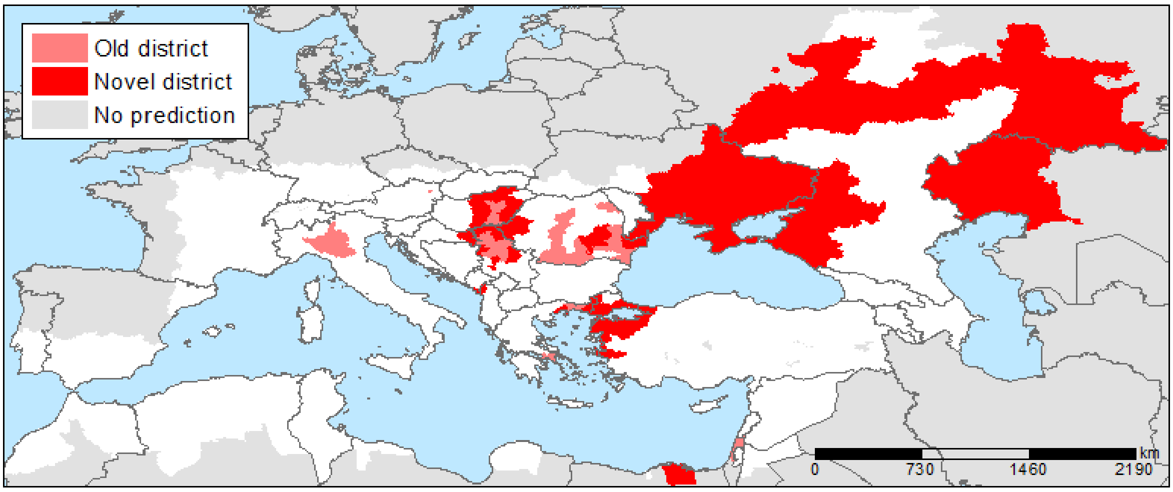

B. Total

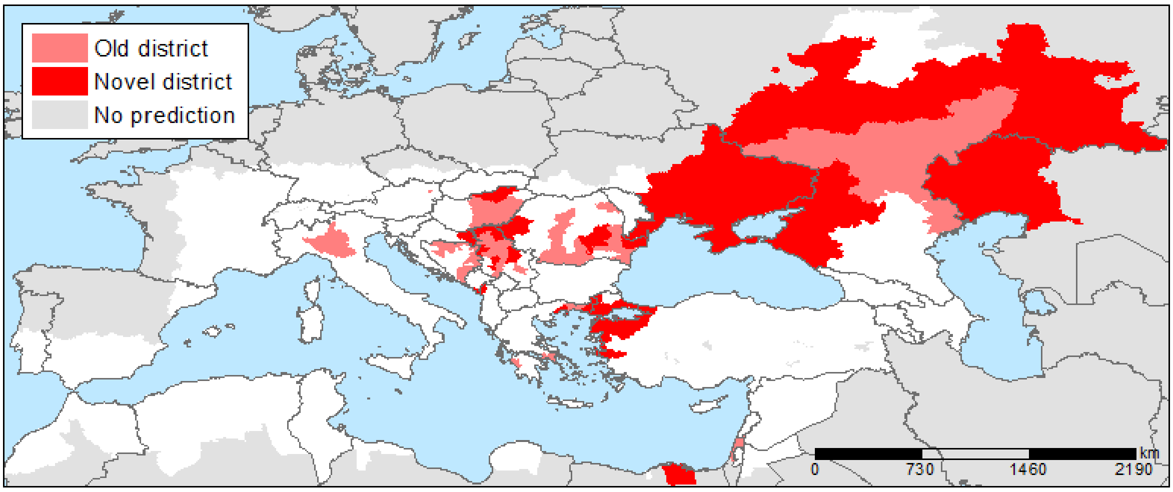

Note: A probable case is any person meeting the clinical criteria AND with at least one of the following two: - an epidemiological link; - a laboratory test for a probable case. A confirmed case is any person meeting laboratory criteria for case confirmation.

Figure 7. Estimated prevalence of West Nile Virus infections in the blood donor population (per 100,000) by districts for 2014 (A) and for 2025 (B).

A:

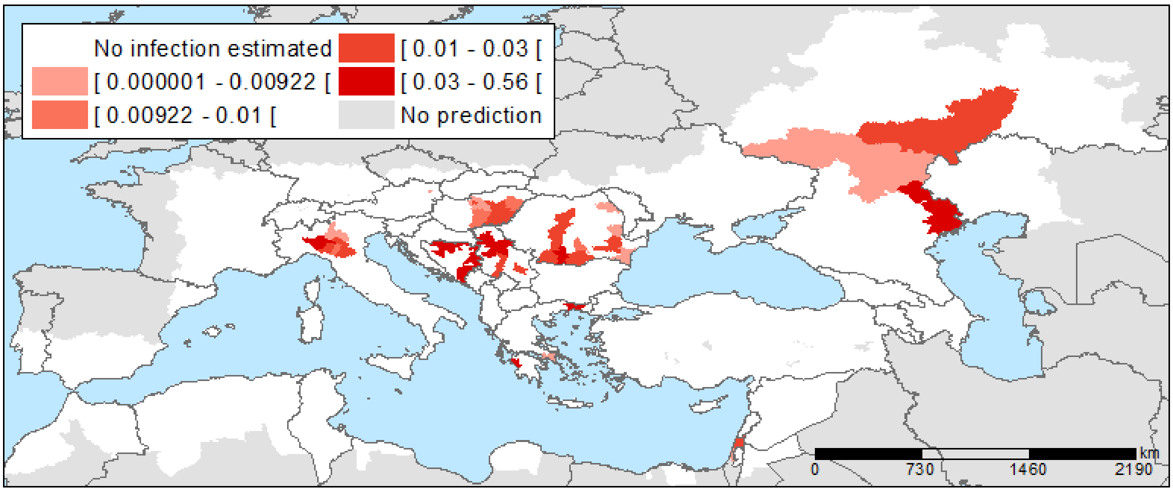

B:

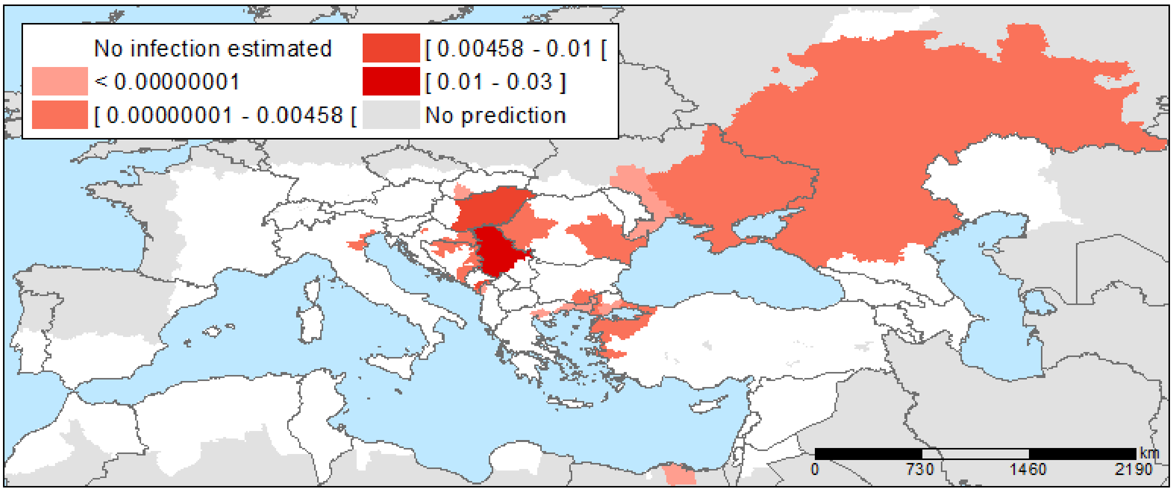

Supplement: Additional file 1: — Peer review reports. (PDF 743 kb) [file 12940_2016_105_MOESM1_ESM.pdf]
